# Supplementary figures and images for: People with more extreme attitudes towards science have self-confidence in their understanding of science, even if this is not justified
Source: PLoS Biol. 2023 Jan 24;21(1):e3001915. doi: 10.1371/journal.pbio.3001915 (PMC10045565; doi:10.1371/journal.pbio.3001915)

A.

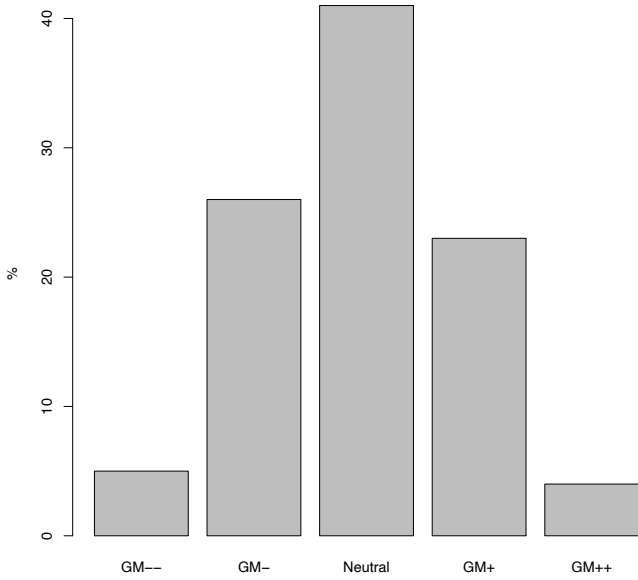

B.

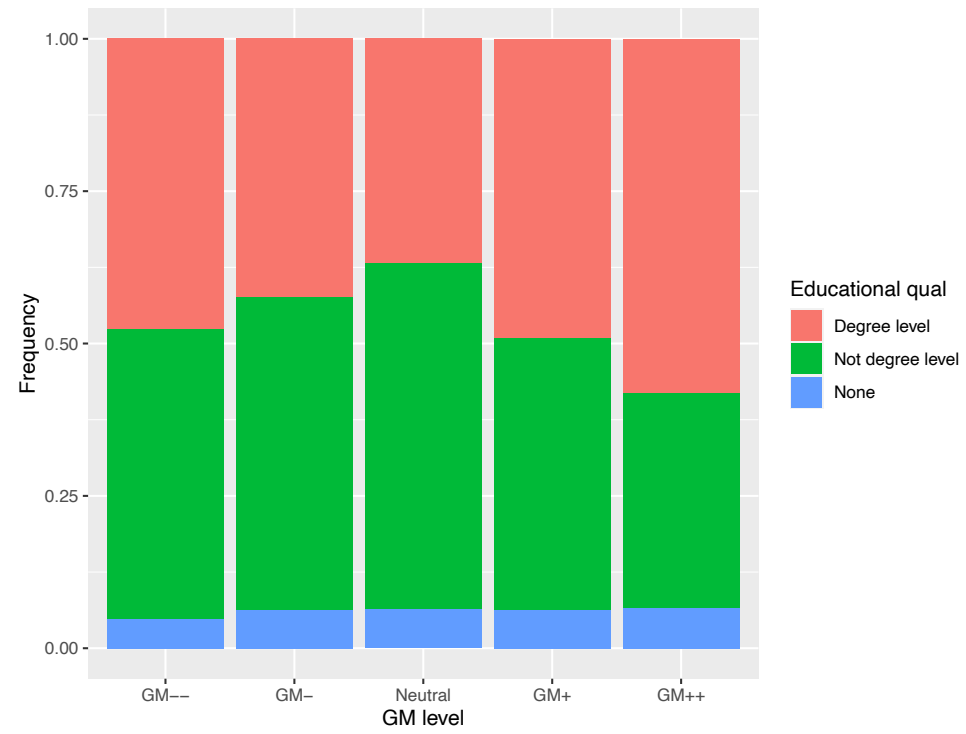

C.

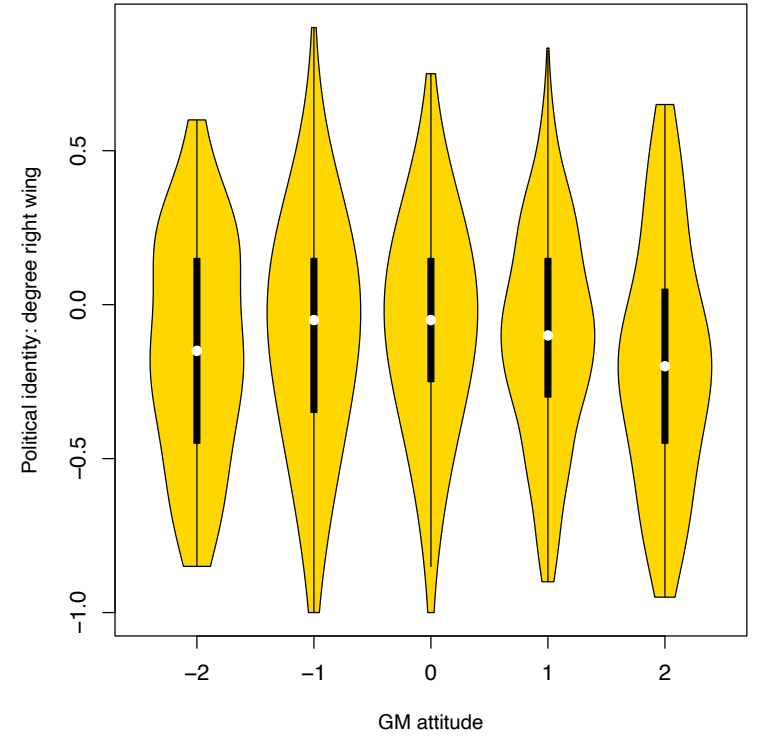

Supplement: S1 Fig — Attitude to GM (a) as a histogram plot, (b) in relation to educational attainment, and (c) in relation to political attitude. All scripts and data are available at doi: 10.5281/zenodo.7289133. (PDF) [file pbio.3001915.s001.pdf]

A.

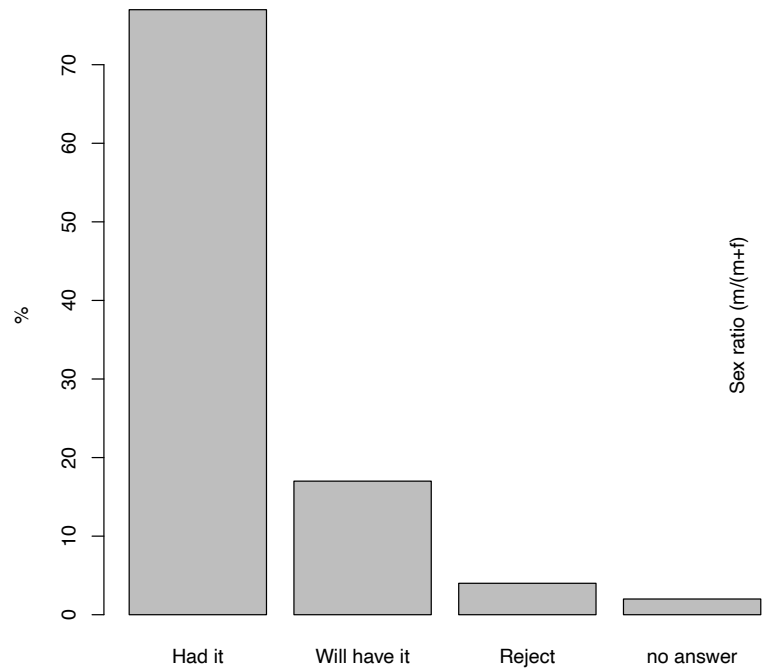

B.

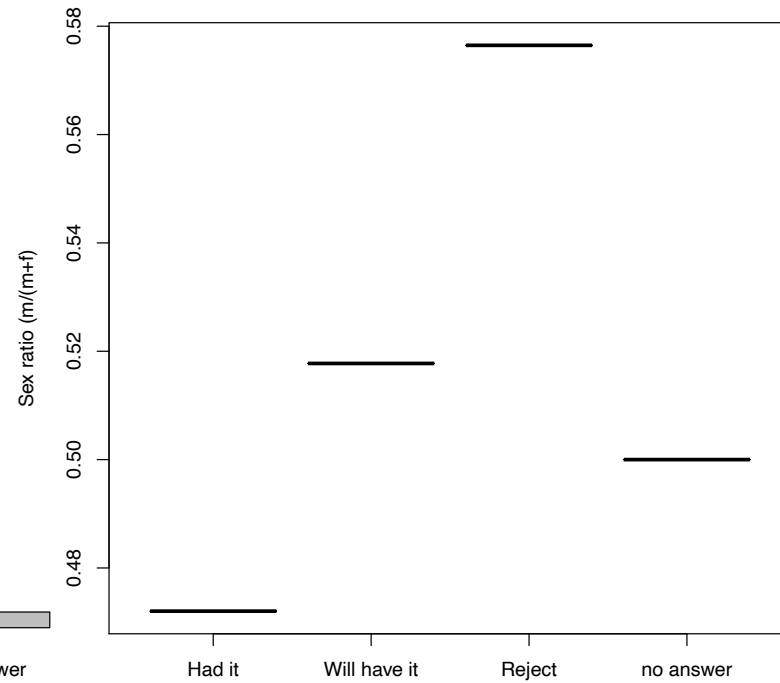

C.

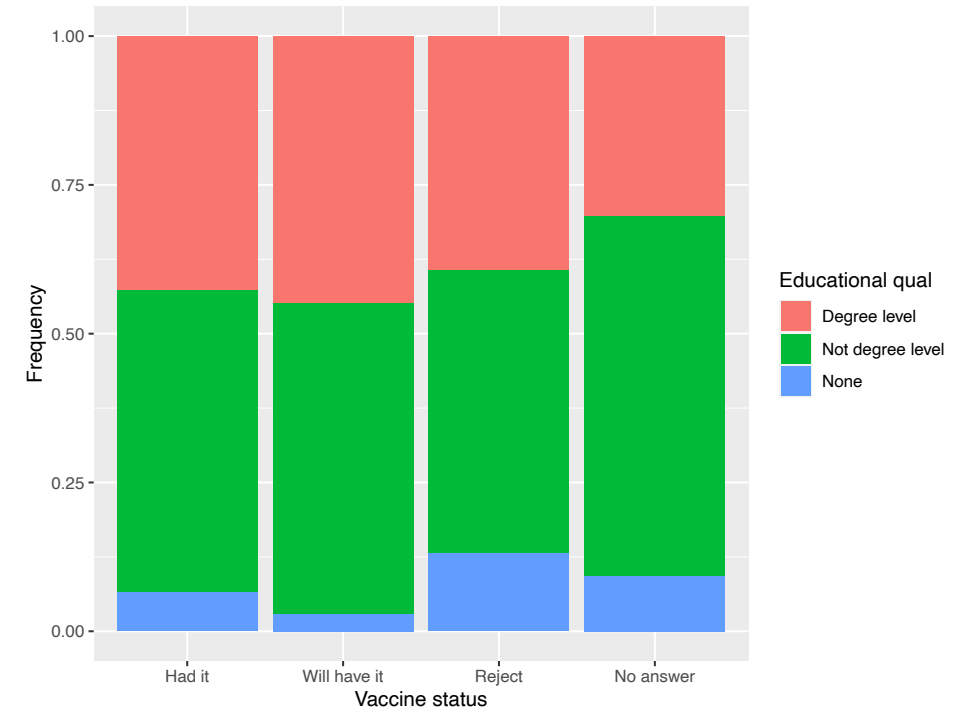

D.

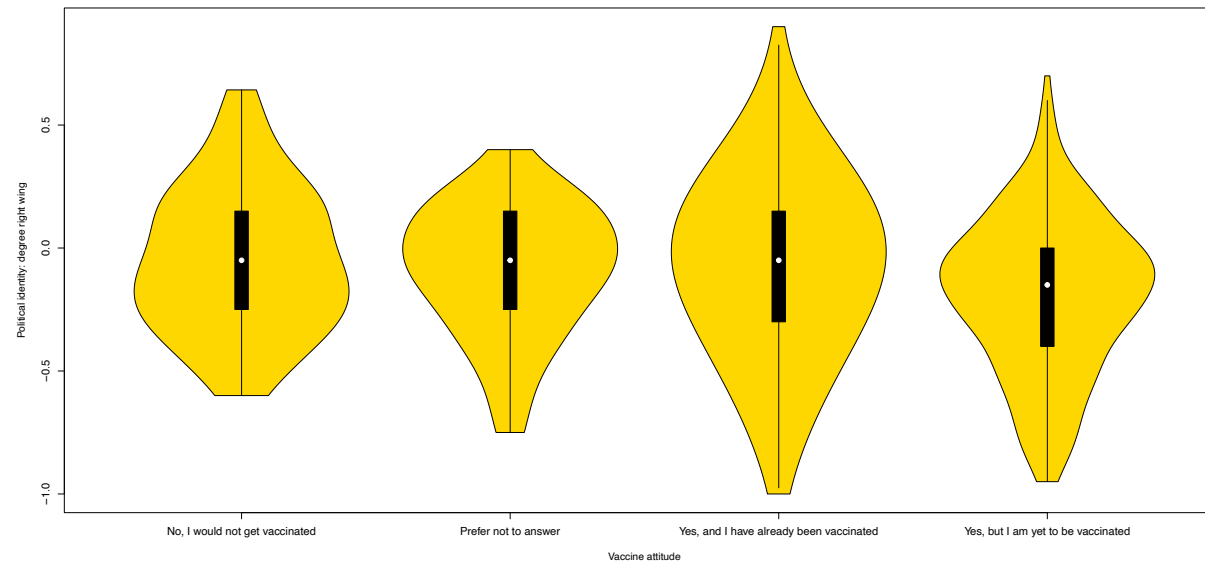

Supplement: S2 Fig — Vaccine attitude (a) as a histogram plot, (b) as related to sex ratio, (c) in relation to educational attainment, and (d) in relation to political attitude. All scripts and data are available at doi: 10.5281/zenodo.7289133. (PDF) [file pbio.3001915.s002.pdf]

Political identity: Right wing degree

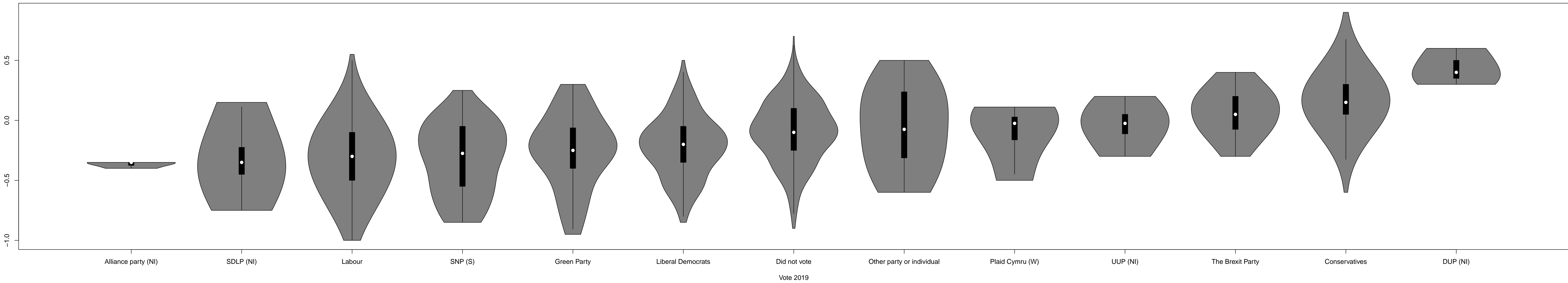

Supplement: S3 Fig — All scripts and data are available at doi: 10.5281/zenodo.7289133. (PDF) [file pbio.3001915.s003.pdf]
