# Supplementary material for: People with more extreme attitudes towards science have self-confidence in their understanding of science, even if this is not justified
Source: PLoS Biol. 2023 Jan 24;21(1):e3001915. doi: 10.1371/journal.pbio.3001915 (PMC10045565; doi:10.1371/journal.pbio.3001915)
Supplement: S1 Materials — All scripts and data are available at doi: 10.5281/zenodo.7289133. (PDF) [file pbio.3001915.s004.pdf]

KANTAR PUBLIC

Public Voice

Technical note on panel survey 11

June 2021

1.1 Objectives

Panel survey 11 (PV11) comprised one survey, for the Genetics Society. The target population was UK individuals aged 18+ and living in residential accommodation.

1.2 Sample and fieldwork design

1.2.1 Sample design

The sample for PV11 was drawn from among the 10,079 respondents to the RS1 and RS2 recruitment surveys who were (i) resident in the UK based on the latest information available, (ii) aged 18+ or of unknown age, and (iii) had joined the Public Voice panel.

The Public Voice panel has been internally stratified with panel members' allocation to one of five 'selection strata' determined by the size of their recruitment survey weight. In general, the larger this weight, the more statistically valuable the panel member but this rule will not hold exactly for every survey. The definition of each selection stratum and the number of panel members in each one is shown in table 1 below.

Table 1 Within-panel stratification details (Target population: UK, 18+)

Selection stratum	Recruitment survey weight range			RS sample size	PV panel sample size	Expected conversion rate if sampled
	Minimum	*Maximum*	*Mean*	*N*	*N*	*%*
1	2.5	>2.5	3.26	680	475	55%
2	1.5	<2.5	1.92	1,503	1,086	57%
3	1	<1.5	1.21	1,673	1,213	60%
4	0.5	<1	0.72	3,438	2,607	62%
5	>0	<0.5	0.27	5,924	4,698	66%
Total			0.85	13,218	10,079	63%

The target respondent sample size for PV11 was 2,000. Following standard practice, a sample was drawn such that (i) the issued sample would be expected to yield at least 2,100 QC-compliant responses, i.e. an overage of >=5% versus the target, and (ii) a reserve sample was available that is one quarter of the size of the issued sample.

Within these constraints – as well as the limitations imposed by the panel sample size in each stratum – a non-linear generalised regression algorithm (operationalised within the MS Excel Solver tool) was used to identify the most 'statistically efficient' sample. For this purpose, statistical efficiency was measured under the simplifying assumption that the only weighting required would be to ensure that

the sum of PV11 respondent weights was equal to the sum of recruitment survey weights with respect to each selection stratum.

In practice, the weighting process is much more complex and the selection stratum is only used for one component of it (to calculate survey design weights). However, it is a reasonable way to optimise a sample design in which sampling fractions vary between but not within these strata. Table 2 shows the sample sizes drawn from each selection stratum.

Table 2 Issued sample sizes in each selection stratum

Selection stratum	Issued sample size	Expected respondent sample size	Reserve sample size
	N	*N*	*N*
**1**	459	251	0
**2**	726	417	213
**3**	598	364	177
**4**	894	564	264
**5**	851	555	248
**Total**	3,528	2,151	902

Before sampling, the panel was sorted within each selection stratum by (i) original recruitment survey stratum, and (ii) household serial number. This approach - combined with a systematic random sampling method - maximises the geodemographic representativeness of any sample drawn from the panel as well as minimising the number of households in which more than one panel member is selected for the same survey.

3,528 of the 4,430 sampled panel members were scheduled to be issued with the remaining 902 allocated to a reserve pool. There was no reserve pool for stratum 1 as all survey-eligible panel members in that stratum (459) were to be issued.

The 3,528 issued panel members were distributed across 3,171 households (1.11 per household).

1.2.2 Fieldwork design

Panel survey 11 was launched with email invitations sent 19th May 2021 and telephone fieldwork starting on 1st June 2021. Of the 3,528 issued cases, only 1,328 (38%) were eligible for inclusion in the telephone fieldwork phase. Inclusion is determined by a mix of strategic and tactical factors: in short, panel members who are statistically more valuable than average *or* are best reached by telephone are included in the telephone fieldwork phase.

A random sample of 786 cases from the reserve pool[1] was issued on 8th June 2021, receiving one email and one text message invitation only (see below for explanation).

All fieldwork was complete by 10th June 2021. The online survey remained open during the telephone interview stage.

Panel survey 11 used the contact design outlined in table 1.2.

[1] Excluding a small number to whom no email or text message could be sent.

All emails contained individualised survey hyperlinks so no login details were required. Additional verification was based on the panel member's birthdate (including year). Where an email address was available, text messages were used only as a supporting communication, sent to non-responders 24

hours after the email and with no embedded survey hyperlink. However, the last text message (contact stage 3) *did* contain a survey hyperlink. Where an email address was not available, all text messages contained a survey hyperlink. The contact management system *sendinblue*[2] was used for all email and text message communications.

A reminder letter - containing survey login details but not a printed individualised survey hyperlink - should have been sent to all non-responders on the 31st May but a mailing error meant that it was not sent in time. This would have been the second letter for the small group for which only an address or landline number was available (and therefore no emails or text messages had been sent).

Because the reminder letter was not sent in time, the majority of the reserve pool needed to be issued just before the end of fieldwork to ensure that the target respondent sample size was achieved.

Those who completed online – and for whom an email or mobile telephone number was available - were sent a thank you email and/or text message together with a £10 e-voucher. Those who completed by telephone – or for whom no email or mobile telephone number was available – were sent a thank you letter, including a £10 shopping voucher card Table 1.2: Contact design, panel survey 11

		CONTACT GROUP				
Day	**Contact stage**	**Email and mobile phone (n = 2,145)**	**Email but no mobile phone (n = 914)**	**Mobile phone but no email (n = 203)**	**Address only or address & landline (n = 176)**	**Phone first [3] (n = 90)**
1	*1*	Email with survey link + supporting text message with no link (24hrs later)	Email with survey link	Text message with survey link	Letter	
5	*2*	Email with survey link + supporting text message with no link (24hrs later)	Email with survey link	Text message with survey link		Advance letter
12	*3*	Email with survey link + supporting text message with survey link (24hrs later)	Email with survey link	Text message with survey link		
13	*	*Not sent*: Letter	*Not sent*: Letter	*Not sent*: Letter	*Not sent*: Letter	

14	*4*	Issue to telephone	Issue to telephone (if landline number)	Issue to telephone	Issue to telephone (if landline number)	Issue to telephone
21	*5*	*Reserve sample only*: Email with survey link + supporting text message with survey link (24hrs later)	*Reserve sample only*: Email with survey link	*Reserve sample only*: Text message with survey link		
23		Close of fieldwork				

[2] https://www.sendinblue.com/

[3] These are panel members who reported in the recruitment survey that they do not use the internet at all *or* do not use it much. They were given the choice of mode. Panel members who have requested first/telephone only contact since then are also included in this group.

1.3 Questionnaire design (panel survey 11)

The questionnaire for panel survey 11 comprised a single survey for the Genetics Society. A draft online questionnaire was supplied by this client and then iteratively amended by the client and the Kantar Public research team. No formal piloting was carried out.

The questionnaire also sought updates of (i) where the panel member lives, (ii) working status, and (iii) contact details. For the most part, the respondents needed only to confirm details already supplied in the recruitment survey but any changes were collected here. A 'current status' data file is kept for all panel members, initially equal to the recruitment survey status but updated where applicable.

1.4 Fieldwork performance

In total, 2,065 questionnaires were completed and passed a QC test. The QC test had two components: (i) the respondent completed the last substantive question, and (ii) the complete questionnaire length was at least 40% of the median for the relevant mode. The shortest completed online questionnaire that passed the QC test was 5:38; for telephone it was 14:48.

The overall conversion rate (the number completing the survey and passing the QC test divided by the number issued for fieldwork) was 48%. However, this was artificially low due to the need to issue a reserve sample. Based on the originally issued sample only, the conversion rate was 52%.

Of the 2,065 respondents, 1,948 (94%) completed the survey online while 117 (6%) completed the survey by telephone interview.

In total, 759 of the 3,528 initially issued panel members were called at least once by a telephone interviewer, although some of these ended up completing the survey online. Of those called at least once, 41% finished with an 'interim' outcome meaning that, technically, more calls could have been made. The mean number of calls made to these numbers was 7.5[2] and the median was 8. Fewer than one in ten (7%) received fewer than five calls, and closer to one in twenty (5%) received fewer than three calls. The overall conversion rate among those called at least once was 16% (119/759) but this includes two 'duplicates' (panel members who completed the survey via both modes due to a lag between online data being captured and it being registered with the sample management tool as a completed questionnaire). Only 117 telephone interviews passed into the final dataset. Table 1.3

[2] Additional calls in the same time block on the same day have been disregarded in this analysis. Time blocks are defined as 'morning' (00:01 to 12:00), "afternoon" (12:01 to 18:00) and "evening" (18:00 to 00:00).

shows the final disposition of all issued cases, disaggregated by sample issue. Table 1.3: Final disposition of all issued cases, survey 11

	Initial issued N	%	Reserve N	%
**Issued**	3,528	100%	786	100%
**Online questionnaire completed and passed QC**	1,717	49%	231	29%
**Telephone questionnaire completed and passed QC**	117	3%	0	0%
**No online completion, issued to telephone, noninterview final outcome**	330	9%	0	0%
**No online completion, issued to telephone, no final outcome**	312	9%	0	0%
**No online completion, not issued to telephone**	935	27%	555	71%

1.5 Response analysis

Given the multi-cohort nature of the *Public Voice* panel, it is not strictly possible to compute an *overall* response rate. However, it is possible to compute cohort-level response rates as the product of (i) the recruitment survey response rate, (ii) the conditional probability of being available for survey 11, given response to the recruitment survey *and* eligibility for survey 11, and (iii) the conditional probability of responding to survey 11, given allocation to survey 11. On this basis, the survey 11 response rate for cohort 1 (RS1 FTFI protocol) was 8.2% (34.8%*49%*48%), for cohort 2 (RS1 ABOS protocol) it was 3.5% (7.2%*69%*63%), and for cohort 3 (RS2) it was 3.5% (9.3%*83%*45%). If these are combined together and weighted on the basis of allocation ratios to survey 11, the overall response rate would be 3.9% but there are other ways of weighting these cohort-level response rates.

One measure of the representativeness of a respondent sample is its 'weight efficiency' after it has been calibrated to a benchmark. In this case, the benchmark is the weighted recruitment survey respondent sample, standing in for the true target population (i.e. all UK-resident individuals aged 18+ living in private accommodation). A perfectly representative sample will have a weight efficiency of 100%, indicating that no variance in response probabilities was observed.

The weight efficiency for the survey 11 dataset was 72%. This means that the confidence intervals for survey 11 estimates were 1.16 times wider than they would have been had the survey 11 respondent sample been perfectly representative of the target population.

The survey 11 weight efficiency of 72% is substantially higher than the equivalent for the recruitment surveys taken together so there was some gain in representativeness between the recruitment surveys and panel survey 11. This was largely due to the sampling protocol adopted (i.e. different sampling fractions applied to each selection stratum). Furthermore, sampling and non-response between the recruitment survey and panel survey 11 reduced the mean number of cases per sample cluster (OA-based areal units for those recruited via the FTFI protocol; addresses for those recruited via the ABOS protocol). This had a positive knock-on effect on the *overall* statistical efficiency of panel survey 11, as measured by the recruitment survey variables. On this basis, the relative overall statistical efficiency of the survey 11 dataset (compared to the recruitment survey dataset) was 177%.

1.6 Weighting

The survey 11 respondent sample was weighted in three stages:

1) For every survey 11 respondent, a *base weight* was calculated that was equal to his/her recruitment survey weight divided by the probability of being sampled for survey 11 (which varied by selection stratum).
2) For every survey 11 respondent, a *propensity score weight* was estimated, as a function of the recruitment survey variables. Technically, this propensity score weight was equal to the estimated odds of being present in the fully weighted recruitment survey dataset *rather than* the base-weighted survey 11 respondent dataset when the latter dataset is added to the former (meaning that survey 11 respondents are present in both datasets). To limit overreliance on the model, the propensity score weight was limited to the inter-95%ile range, and the value of the product of the base and propensity score weights was trimmed to have a maximum equal to the 98th percentile value. This was used as weight (2).
3) Using weight (2) as a starting point, the survey 11 respondent sample was calibrated to the weighted ONS *Labour Force Survey* of October to December 2020 with respect to sex*age group, region, birth country, and highest educational level. The simple raking algorithm was used for this step.

The selection of predictor variables for the logistic regression model (step 2) was an iterative process. The first step was to carry out chi-square tests to compare the respective recruitment survey data distributions of (i) survey 11 respondents and (ii) the full recruitment survey dataset, using the previously described base weight (1) for the survey 11 respondents, and the recruitment survey weight for the full recruitment survey dataset.

Any variable with a chi-square test p score below 1% was treated as a candidate predictor variable[3]. Fifty-two of the 103 variables passed this filter. These variables were included as main effects in the first (complete-case) iteration of the logistic regression model in which dataset identity (recruitment survey dataset or survey 11 respondent dataset) was the dependent variable. Respondent sex and age group were included by default (age group was one of the initial 52).

Only 18 of these 53 variables had Wald F p scores below 20%, suggesting independent predictive value in the model. A second main effects model was estimated based only on these variables and then a third and final model was estimated based on the 12 variables with Wald F p scores under 5% in the second model (plus respondent sex). The vast majority of survey 11 respondents had data for all 13 variables and received a non-response weight. For the rest, a non-response weight was estimated using the SPSS MULTIPLE IMPUTATION chained equation FCS algorithm and based on however much data was available from among the predictor variables in the substantive model.

The recruitment survey variables included in the final model of survey 11 response were: (i) whether the individual has an email address that is checked regularly, (ii) whether he/she has some wearable technology (e.g. a fitbit), (iii) whether he/she has household access to a Smart TV, (iv) his/her opinion about spending on benefits, (v) whether he/she has access to a PC, (vi) his/her opinion on banning some internet sites, (vii) age group, (viii) whether he/she has done any voluntary work recently, (ix) the extent to which he/she thinks of themself as a quiet person, (x) region, (xi) his/her opinion about 'big business', (xii) frequency of using the internet, and (xiii) sex.

Table 1.4 shows the relative survey 11 response probability for each category of each variable in the model. The mean response probability is indexed at 100. The strongest predictor of participation in survey 11 was use of email: those regularly checking their account had a survey 11 response index of 102 but those without such an account had a survey 11 response index of only 61.

Table 1.4: Panel survey 11 relative response probabilities (13 variables in response model)

Variable from RS	Category	Recruitment survey n (eligible)	Relative panel survey 11 response probability (indexed at mean 100)
	All	13,218	100

[3] A p score of <1% means that there is a <1% probability of a difference of the observed magnitude occurring by chance *if* the two sampling procedures would, on average, produce the same data distributions.

**Whether has email address that is regularly checked**	No	1,065	61
	Yes	12,119	102
**Wearable technology**	No	9,977	97
	Yes	3,241	110
**Smart TV**	No	5,830	96
	Yes	7,388	103
**Opinion about benefits spending**	Reduce spending on benefits	3323	96
	Keep spending on benefits at the same level as now	5421	99
	Increase spending on benefits	3996	107
**PC**	No	2,938	82
	Yes	10,280	104
**"Lots of internet sites should be banned"**	Strongly agree	2,404	88
	Tend to agree	3,311	99
	Neither agree nor disagree	3,619	99
	Tend to disagree	2,199	106
	Strongly disagree	1,418	116
**Ten year age band**	16-24	1,226	89
	25-34	2,319	100
	35-44	1,992	100
	45-54	2,122	99
	55-64	2,294	107
	65-74	2,122	108

	75+	1,133	86
**Voluntary work**	In the last 4 weeks	1,845	107
	Not in the last 4 weeks but in the last 12 months	3,049	107
	Not in the last 12 months	8,212	96
**“I am someone who tends to be quiet”**	Disagree strongly	1,932	84
	Disagree a little	3,025	105
	Neutral; no opinion	1,687	93
	Agree a little	4,171	104
	Agree strongly	2,307	107
**Region**	NE England	556	115
	NW England	1,467	102
	Yorkshire & Humberside	1,112	97
	E Midlands	995	104
	W Midlands	1,059	96
	E England	1,252	95
	London	1,707	97
	SE England	1,879	113
	SW England	1,303	103
	Northern Ireland	275	76
	Scotland	1,027	87
	Wales	586	86
	Strongly agree	3894	105

**"Big business benefits owners at the expense of the people who work for them"**	Tend to agree	4903	101
	Neither agree nor disagree	2640	90
	Tend to disagree	1145	108
	Strongly disagree	364	97
**Self-reported frequency of using the internet**	Five hours or more every day	1,130	98
	Three hours or more but less than five hours every day	1,941	108
	Two hours or more but less than three hours every day	2,962	104
	One hour or more but less than two hours every day	3,403	108
	Less than one hour every day	1,153	93
	Most days	1,252	84
	A few times a week	477	80
	Less often	312	76
	Never	570	72
**Sex**	Male	6,194	102
	Female	6,954	99
	Identify differently	63	137

Weight (2) compensates for survey 11 non-response bias effectively. However, a calibration weight (3) was generated to fulfil the general protocol for Public Voice surveys that sex/age, region, highest educational qualification and birth country distributions should be exactly aligned with national statistics.

The calibration step (3) did not noticeably improve the alignment between the weighted survey 11 respondent dataset and the weighted recruitment survey dataset but it was already close after step (2) of the process. Based on the standard set of 377 category-level proportions, the median difference between the weighted survey 11 respondent dataset and the weighted recruitment survey dataset was only 0.7 percentage points.

Table 1.5 shows the calibration matrix that was used for survey 11, derived from the ONS *Labour Force Survey* of October through December 2020 but with some adjustments to reflect minor differences between the LFS and Public Voice variables.

Table 1.5: ONS *Labour Force Survey* population estimates, October through December 2020, UK adults aged 18+

Variable from RS	Category	% of population
	All	100.0
Sex/age group	Male 18-24	5.3
	Male 25-34	8.6
	Male 35-44	8.0
	Male 45-54	8.3
	Male 55-64	7.8
	Male 65-74	6.1
	Male 75+	4.7
	Female 18-24	5.1
	Female 25-34	8.5
	Female 35-44	8.2
	Female 45-54	8.5
	Female 55-64	8.2
	Female 65-74	6.6
	Female 75+	5.9
	*Other	0.2
Region	NE England	4.0
	NW England	10.9
	Yorkshire & The Humber	8.2
	E Midlands	7.2
	W Midlands	8.8

	E England	9.5
	London	13.3
	SE England	13.7
	SW England	8.6
	Scotland	8.4
	Wales	4.7
	Northern Ireland	2.7
**Highest education level**	Degree level qualifications, aged 16-69	30.3
	Lower qualifications, aged 1669	47.1
	No qualifications, aged 16-69	5.6
	Aged 70+	17.0
**UK birth/citizenship status**	UK born	86.0
	Not UK born	14.0

* Declaration of sex as 'identify differently' fixed at recruitment survey weighted level

1.7 Data quality standards

The survey 11 clients requested that respondents be included in the survey dataset so long as they completed the last question in the client questionnaire module *and* the overall questionnaire length was at least 40% of the median by mode. Forty-five respondents (2%) were excluded for going too quickly through the questionnaire.

The length criterion meant an overall minimum of 5 minutes 38 seconds for the online survey (median = 14 minutes 11 seconds), and a minimum of 14 minutes 48 seconds for the telephone survey (median = 27 minutes 26 seconds).

1.8 Online (CAWI) Questionnaire

Q1101 - Intro: **Text**

The following section of questions is asked on behalf of the Genetics Society. It covers a range of topics relating to your views on new developments in the field of science and your understanding of certain scientific topics.

Q1111 - Stories: **Single coded**

Thinking of the stories about science you see or hear in the news, which of the following statements would you say best describes you?

1	I usually understand what they are talking about
2	I sometimes understand what they are talking about
3	I usually do not understand what they are talking about
4	I don’t see or hear science news stories

Q1112 - Informed: **Single coded**

How well informed do you feel, if at all, about science, and scientific research and developments?

1	Very well informed
2	Fairly well informed
3	Not very well informed
4	Not at all informed
999	Don't know **Fixed *Exclusive*

Q1113 - DNA: **Single coded**

We'd now like to ask you about your understanding of different scientific terms that are used in news stories dealing with medical research.
First, when you hear the term DNA, how would you rate your understanding of what the term means?

1	Very good
2	Good
3	Some understanding
4	Have heard the term but have little understanding of what it means
5	Have not heard the term

Q1114 - GM: **Single coded**

Next, when you hear the term GM or genetically modified, how would you rate your understanding of what the term means?

1 Very good
2 Good
3 Some understanding
4 Have heard the term but have little understanding of what it means 5 Have not heard the term

Q1115 - NaturalSelection: **Single coded**

Next, when you hear the term natural selection, how would you rate your understanding of what the term means?

1 Very good
2 Good
3 Some understanding
4 Have heard the term but have little understanding of what it means 5 Have not heard the term

Q1116 - PCR: **Single coded**

Next, when you hear the term PCR, how would you rate your understanding of what the term means?

1 Very good
2 Good
3 Some understanding
4 Have heard the term but have little understanding of what it means 5 Have not heard the term

Ask only if **Q1113 - DNA**,1,2,3,4

Q1117 - UndDNA: **Single coded**

Now, thinking about the news and stories you have heard since the start of the pandemic, do you feel that your understanding of DNA has…

1 Increased
2 Stayed the same
3 Decreased
999 Don't know **Fixed *Exclusive*

Ask only if **Q1114 - GM**,1,2,3,4

Q1118 - UndGM: **Single coded**

Now, thinking about the news and stories you have heard since the start of the pandemic, do you feel that your understanding of GM has…

1	Increased
2	Stayed the same
3	Decreased
999	Don't know **Fixed *Exclusive*

Ask only if **Q1115 - NaturalSelection**,1,2,3,4

Q1119 - UndNaturalSelection: **Single coded**

Now, thinking about the news and stories you have heard since the start of the pandemic, do you feel that your understanding of natural selection has…

1	Increased
2	Stayed the same
3	Decreased
999	Don't know **Fixed *Exclusive*

Ask only if **Q1116 - PCR**,1,2,3,4

Q11110 - UndPCR: **Single coded**

Now, thinking about the news and stories you have heard since the start of the pandemic, do you feel that your understanding of PCR has…

1	Increased
2	Stayed the same
3	Decreased
999	Don't know **Fixed *Exclusive*

Q11111 - BeforePand: **Matrix**

And now, thinking back to before the start of the pandemic, to what extent would you have agreed or disagreed with the following statements at that time?

		Strongly agree	Agree	Neither agree or disagree	Disagree	Strongly disagree
		1	2	3	4	5
Many claims about the benefits of modern genetic science are greatly exaggerated.	1	❍	❍	❍	❍	❍

Those in charge of new developments in genetic science cannot be trusted to act in society's interests.	2	❍	❍	❍	❍	❍
On balance, the advantages of genetically modified (GM) foods outweigh any dangers.	3	❍	❍	❍	❍	❍

Q11112 - ViewChange1: **Single coded**

How do you think your views have changed in the last year? Thinking about the statement "Many claims about the benefits of modern genetic science are greatly exaggerated" would you say that you have…

1	Become more likely to agree with this
2	Not changed your opinion
3	Become more likely to disagree with this
999	Don't know **Fixed *Exclusive*

Q11113 - ViewChange2: **Single coded**

How do you think your views have changed in the last year? Thinking about the statement "Those in charge of new developments in genetic science cannot be trusted to act in society's interests" would you say that you have…

1	Become more likely to agree with this
2	Not changed your opinion
3	Become more likely to disagree with this
999	Don't know **Fixed *Exclusive*

Q11114 - ViewChange3: **Single coded**

How do you think your views have changed in the last year? Thinking about the statement "On balance, the advantages of genetically modified (GM) foods outweigh any dangers" would you say that you have…

1	Become more likely to agree with this
2	Not changed your opinion
3	Become more likely to disagree with this
999	Don't know **Fixed *Exclusive*

Q11115 - Statements: **Matrix**

For each of the following statements, please say whether you think it is definitely true, probably true, probably false or definitely false.

		Definitely true	Probably true	Probably false	Definitely false	Don't know *Fixed *Exclusive
		1	2	3	4	999
All plants and animals have DNA	1	❍	❍	❍	❍	❍
The oxygen we breathe comes from plants	2	❍	❍	❍	❍	❍
The cloning of living things produces genetically identical copies	3	❍	❍	❍	❍	❍
By eating a genetically modified fruit, a person's genes could also become modified	4	❍	❍	❍	❍	❍
All radioactivity is human-made	5	❍	❍	❍	❍	❍
It is the mother's genes that determine the sex of the child	6	❍	❍	❍	❍	❍
Electrons are smaller than atoms	7	❍	❍	❍	❍	❍
Tomatoes do not naturally contain genes; genes are only found in genetically modified tomatoes	8	❍	❍	❍	❍	❍
Dinosaurs and humans share a common ancestor	9	❍	❍	❍	❍	❍
The spread of new variants of viruses can occur through natural selection	10	❍	❍	❍	❍	❍
COVID-19 is caused by bacteria	11	❍	❍	❍	❍	❍
Viruses are smaller than bacteria	12	❍	❍	❍	❍	❍

**Q11116 - AttGenetics:**	**Single coded**

On balance, which of the following best describes your attitude to the scientific study of genetics at present?

1	Very positive
2	Slightly positive
3	Undecided
4	Slightly negative
5	Very negative
6	I don’t know enough to form a judgement

**Q11117 - Optimism:**	**Single coded**

How optimistic are you about the possibility of improved healthcare as a result of genetic research?

1	Very optimistic,
2	Somewhat optimistic,
3	Not too optimistic, OR,
4	Not at all optimistic

**Q11118 - SciRelationship:**	**Single coded**

Which of these statements best describes your relationship with science?

1	I feel connected with science – I actively seek out science news, events, activities or entertainment
2	I’m interested in science, but I don’t make a special effort to keep informed
3	Science is not for me

**Q11119 - ThreeMonths:**	**Multi coded**

Which, if any, of these have you seen, read or heard about over the last three months?

1	Services offering online genetic profiling for individuals, such as “23andMe” or “AncestryDNA” 2
	New techniques for scientists to be able to edit the genomes of plants, animals or humans
3	PCR testing for COVID-19
4	Natural selection operating on viruses
5	Any negative or concerning stories about genomic research or medicine
6	None of the above **Exclusive*

**Q11120 - WhoTrust:**	**Multi coded**

Who would you trust to provide accurate and reliable information about COVID-

.9?

PLEASE SELECT ALL THAT APPLY.

1	Work colleagues
2	The government’s scientific advisers
3	The government
4	Celebrities and public figures
5	Not-for-profit organisations/charities
6	NHS spokesperson
7	Research Scientist / Universities
8	Family / friends
9	None of these **Exclusive*
999	Don't know **Fixed *Exclusive*

Q11121 - WhichTrust: **Multi coded**

And which of these sources would you trust to provide accurate and reliable information about COVID-19?

PLEASE SELECT ALL THAT APPLY.

1	A Government website
2	TV news programmes
3	Topical TV shows (e.g. ‘The One show’ or ‘Have I got news for you’)
4	Celebrities and Influencers on social media platforms like Facebook or Twitter
5	Organisations e.g. charities/universities/professional bodies on Social media
6	Scientists on Social media
7	Other individuals on Social media
8	YouTube
9	Websites which focus on this topic
10	Newspapers
11	Online-only news sites (e.g. Huffington Post, Google News)
12	Online news sites of traditional media outlets (e.g. BBC news, ITV news etc)
13	BBC National radio (e.g. BBC Radio1)
14	BBC local radio (e.g. BBC Wiltshire)
15	Commercial radio stations (e.g. Capital FM)
16	None of these **Exclusive*
999	Don't know **Fixed *Exclusive*

Q11122 - HeardGenes: **Single coded**

Over the last few months, how much, if anything, have you heard or read about issues to do with genes and genetics?

1	A great deal
2	Quite a lot
3	A small amount
4	Not very much
5	Not at all

Q11123 - ThoughtGenes: **Single coded**

Over the past few months, how much, if at all, have you thought about issues to do with genes and genetics?

1	A great deal
2	Quite a lot
3	A small amount
4	Not very much
5	Not at all

Q11124 - TheseDays: **Single coded**

Which of the following statements do you most agree with? These days I hear and see …

1	... far too much information about science
2	… too much information about science
3	… the right amount of information about science
4	… too little information about science
5	… far too little information about science

Scripter notes: If respondent tries to click through without answering, display message saying 'Please select one of the answers before moving to the next question'. If they try to click through without answering for a second time, then allow them to move on without inputting an answer

Q11125 - TrustSci: **Single coded**

Not back

In general, would you say you distrust or trust scientists?

Normal

1	Completely distrust
2	Partially distrust
3	Neither distrust nor trust
4	Partially trust
5	Completely trust
6	Not applicable / No Opinion

Q11126 - TrustGenet: **Single coded**

In general, would you say you distrust or trust geneticists?

1	Completely distrust
2	Partially distrust
3	Neither distrust nor trust
4	Partially trust
5	Completely trust
6	Not applicable / No Opinion

Q11127 - TrustGeol: **Single coded**

In general, would you say you distrust or trust geologists?

Normal

1	Completely distrust
2	Partially distrust
3	Neither distrust nor trust
4	Partially trust
5	Completely trust
6	Not applicable / No Opinion

**Q11128 - PandTrustSci:**	**Single coded**

Would you say you now trust scientists more, less, or about the same as you did at the start of the pandemic?

1	Trust them much more
2	Trust them a little more
3	About the same
4	Trust them a little less
5	Trust them much less
999	Don't know **Fixed *Exclusive*

**Q11129 - PandTrustGenet:**	**Single coded**

Would you say you now trust geneticists more, less, or about the same as you did at the start of the pandemic?

1	Trust them much more
2	Trust them a little more
3	About the same
4	Trust them a little less
5	Trust them much less
999	Don't know **Fixed *Exclusive*

**Q11130 - PandTrustGeol:**	**Single coded**

Would you say you now trust geologists more, less, or about the same as you did at the start of the pandemic?

1	Trust them much more
2	Trust them a little more
3	About the same
4	Trust them a little less
5	Trust them much less
999	Don't know **Fixed *Exclusive*

**Q11131 - Pfiz:**	**Single coded**

Would you say you now trust pharmaceutical companies, e.g. Pfizer, more, less or about the same as you did at the start of the pandemic?

1	Trust them much more
2	Trust them a little more
3	About the same
4	Trust them a little less
5	Trust them much less
999	Don't know **Fixed *Exclusive*

**Q11132 - Glaxo:**	**Single coded**

Would you say you now trust pharmaceutical companies, e.g. GlaxoSmithKline, more, less or about the same as you did at the start of the pandemic?

1	Trust them much more
2	Trust them a little more
3	About the same
4	Trust them a little less
5	Trust them much less
999	Don't know **Fixed *Exclusive*

**Q11133 - HadCov:**	**Single coded**

Do you think that you have, or have had, COVID-19?

1	Yes, confirmed by a positive test
2	Yes, suspected by a healthcare professional but not tested
3	Yes, my own suspicions
4	No
997	Prefer not to answer **Fixed *Exclusive*

**Q11134 - CovVac:**	**Single coded**

If you were offered a COVID-19 vaccine would you take it?

1	Yes, and I have already been vaccinated
2	Yes, but I am yet to be vaccinated
3	No, I would not get vaccinated
997	Prefer not to answer **Fixed *Exclusive*

1.9 Telephone (CATI) questionnaire

Q1101 - Intro: **Text**

READ OUT: The following section of questions is asked on behalf of the Genetics Society. It covers a range of topics relating to your views on new developments in the field of science and your understanding of certain scientific topics.

Q1111 - Stories: **Single coded**

Thinking of the stories about science you see or hear in the news, which of the following statements would you say best describes you? *READ OUT*

1	I usually understand what they are talking about
2	I sometimes understand what they are talking about
3	I usually do not understand what they are talking about
4	SPONTANEOUS: I don't see or hear science news stories

Q1112 - Informed: **Single coded**

How well informed do you feel, if at all, about science, and scientific research and developments? *READ OUT*

1	Very well informed
2	Fairly well informed
3	Not very well informed
4	Not at all informed
999	Don't know **Fixed *Exclusive*

Q1113 - DNA: **Single coded**

We'd now like to ask you about your understanding of different scientific terms that are used in news stories dealing with medical research.
First, when you hear the term DNA, how would you rate your understanding of what the term means? *READ OUT*

1	Very good
2	Good
3	Some understanding
4	Have heard the term but have little understanding of what it means
5	Have not heard the term

Q1114 - GM: **Single coded**

Next, when you hear the term GM or genetically modified, how would you rate your understanding of what the term means? *READ OUT*

1 Very good
2 Good
3 Some understanding
4 Have heard the term but have little understanding of what it means 5 Have not heard the term

Q1115 - NaturalSelection: **Single coded**

Next, when you hear the term natural selection, how would you rate your understanding of what the term means? *READ OUT*

1 Very good
2 Good
3 Some understanding
4 Have heard the term but have little understanding of what it means
5 Have not heard the term

Q1116 - PCR: **Single coded**

Next, when you hear the term PCR, how would you rate your understanding of what the term means? *READ OUT*

1 Very good
2 Good
3 Some understanding
4 Have heard the term but have little understanding of what it means 5 Have not heard the term

Ask only if **Q1113 - DNA**,1,2,3,4

Q1117 - UndDNA: **Single coded**

Now, thinking about the news and stories you have heard since the start of the pandemic, do you feel that your understanding of DNA has… *READ OUT*

1	Increased
2	Stayed the same
3	Decreased
999	Don't know **Fixed *Exclusive*

Ask only if **Q1114 - GM**,1,2,3,4

Q1118 - UndGM: **Single coded**

Now, thinking about the news and stories you have heard since the start of the pandemic, do you feel that your understanding of GM has… *READ OUT*

1	Increased
2	Stayed the same
3	Decreased
999	Don't know **Fixed *Exclusive*

Ask only if **Q1115 - NaturalSelection**,1,2,3,4

Q1119 - UndNaturalSelection: **Single coded**

Now, thinking about the news and stories you have heard since the start of the pandemic, do you feel that your understanding of natural selection has… *READ OUT*

1	Increased
2	Stayed the same
3	Decreased
999	Don't know **Fixed *Exclusive*

Ask only if **Q1116 - PCR**,1,2,3,4

Q11110 - UndPCR: **Single coded**

Now, thinking about the news and stories you have heard since the start of the pandemic, do you feel that your understanding of PCR has… *READ OUT*

1	Increased
2	Stayed the same
3	Decreased
999	Don't know **Fixed *Exclusive*

Q11111 - BeforePand: **Matrix**

And now, thinking back to before the start of the pandemic, to what extent would you have agreed or disagreed with the following statements at that time?
INTERVIEWER INSTRUCTION: READ OUT EACH OF THE STATEMENTS
READ THE ANSWER CHOICES AS NECESSARY AFTER THE FIRST TIME

		Strongly agree	Agree	Neither agree or disagree	Disagree	Strongly disagree
		1	2	3	4	5
Many claims about the benefits of modern genetic science are greatly exaggerated.	1	❍	❍	❍	❍	❍
Those in charge of new developments in genetic science cannot be trusted to act in society's interests.	2	❍	❍	❍	❍	❍
On balance, the advantages of genetically modified (GM) foods outweigh any dangers.	3	❍	❍	❍	❍	❍

Q11112 - ViewChange1: **Single coded**

How do you think your views have changed in the last year? Thinking about the statement "Many claims about the benefits of modern genetic science are greatly exaggerated" would you say that you have… *READ OUT*

1	Become more likely to agree with this
2	Not changed your opinion
3	Become more likely to disagree with this
999	Don't know **Fixed *Exclusive*

Q11113 - ViewChange2: **Single coded**

How do you think your views have changed in the last year? Thinking about the statement "Those in charge of new developments in genetic science cannot be trusted to act in society's interests" would you say that you have… *READ OUT*

1	Become more likely to agree with this
2	Not changed your opinion
3	Become more likely to disagree with this
999	Don't know **Fixed *Exclusive*

Q11114 - ViewChange3: **Single coded**

How do you think your views have changed in the last year? Thinking about the statement "On balance, the advantages of genetically modified (GM) foods outweigh any dangers" would you say that you have… *READ OUT*

1	Become more likely to agree with this
2	Not changed your opinion
3	Become more likely to disagree with this
999	Don't know **Fixed *Exclusive*

Q11115 - Statements: **Matrix**

For each of the following statements, please say whether you think it is definitely true, probably true, probably false or definitely false.
INTERVIEWER INSTRUCTION: READ OUT EACH OF THE STATEMENTS
READ THE ANSWER CHOICES AS NECESSARY AFTER THE FIRST TIME

		Definitely true	Probably true	Probably false	Definitely false	Don't know *Fixed *Exclusive
		1	2	3	4	999
All plants and animals have DNA	1	❍	❍	❍	❍	❍
The oxygen we breathe comes from plants	2	❍	❍	❍	❍	❍
The cloning of living things produces genetically identical copies	3	❍	❍	❍	❍	❍
By eating a genetically modified fruit, a person's genes could also become modified	4	❍	❍	❍	❍	❍
All radioactivity is human-made	5	❍	❍	❍	❍	❍
It is the mother's genes that determine the sex of the child	6	❍	❍	❍	❍	❍
Electrons are smaller than atoms	7	❍	❍	❍	❍	❍
Tomatoes do not naturally contain genes; genes are only found in genetically modified tomatoes	8	❍	❍	❍	❍	❍
Dinosaurs and humans share a common ancestor	9	❍	❍	❍	❍	❍
The spread of new variants of viruses can occur through natural selection	10	❍	❍	❍	❍	❍
COVID-19 is caused by bacteria	11	❍	❍	❍	❍	❍
Viruses are smaller than bacteria	12	❍	❍	❍	❍	❍

Q11116 - AttGenetics: **Single coded**

On balance, which of the following best describes your attitude to the scientific study of genetics at present?
READ OUT

1	Very positive
2	Slightly positive
3	Undecided
4	Slightly negative
5	Very negative
6	I don't know enough to form a judgement

**Q11117 - Optimism:**	**Single coded**

How optimistic are you about the possibility of improved healthcare as a result of genetic research? *READ OUT*

1	Very optimistic
2	Somewhat optimistic
3	Not too optimistic
4	Not at all optimistic

**Q11118 - SciRelationship:**	**Single coded**

Which of these statements best describes your relationship with science? *READ OUT*

1 I feel connected with science – I actively seek out science news, events, activities or entertainment 2 I'm interested in science, but I don't make a special effort to keep informed 3 Science is not for me

**Q11119 - ThreeMonths:**	**Multi coded**

Which, if any, of these have you seen, read or heard about over the last three months? *READ OUT*

1 Services offering online genetic profiling for individuals, such as "23andMe" or "AncestryDNA" 2 New techniques for scientists to be able to edit the genomes of plants, animals or humans

3	PCR testing for COVID-19
4	Natural selection operating on viruses
5	Any negative or concerning stories about genomic research or medicine
6	None of the above **Exclusive*

**Q11120 - WhoTrust:**	**Multi coded**

Who would you trust to provide accurate and reliable information about COVID-19? Please choose all that apply… *READ OUT*

1	Work colleagues
2	The government’s scientific advisers
3	The government
4	Celebrities and public figures
5	Not-for-profit organisations/charities
6	NHS spokesperson
7	Research Scientist / Universities
8	Family / friends
9	None of these **Exclusive*
999	Don't know **Fixed *Exclusive*

Q11121 - WhichTrust: **Multi coded**

And which of these sources would you trust to provide accurate and reliable information about COVID-19? Please choose all that apply… *READ OUT*

1	A Government website
2	TV news programmes
3	Topical TV shows (e.g. ‘The One show’ or ‘Have I got news for you’)
4	Celebrities and Influencers on social media platforms like Facebook or Twitter
5	Organisations e.g. charities/universities/professional bodies on Social media
6	Scientists on Social media
7	Other individuals on Social media
8	YouTube
9	Websites which focus on this topic
10	Newspapers
11	Online-only news sites (e.g. Huffington Post, Google News)
12	Online news sites of traditional media outlets (e.g. BBC news, ITV news etc)
13	BBC National radio (e.g. BBC Radio1)
14	BBC local radio (e.g. BBC Wiltshire)
15	Commercial radio stations (e.g. Capital FM)
16	None of these **Exclusive*

999	Don't know **Fixed *Exclusive*

Q11122 - HeardGenes:	Single coded

Over the last few months, how much, if anything, have you heard or read about issues to do with genes and genetics?
READ OUT

1	A great deal
2	Quite a lot
3	A small amount
4	Not very much
5	Not at all

Q11123 - ThoughtGenes:	Single coded

Over the past few months, how much, if at all, have you thought about issues to do with genes and genetics?
READ OUT

1	A great deal
2	Quite a lot
3	A small amount
4	Not very much
5	Not at all

Q11124 - TheseDays: Single coded

Which of the following statements do you most agree with?
These days you hear and see … *READ OUT*

1	... far too much information about science
2	… too much information about science
3	… the right amount of information about science
4	… too little information about science
5	… far too little information about science

Q11125 - TrustSci: Single coded

In general, would you say you distrust or trust scientists? *READ OUT*

1	Completely distrust
2	Partially distrust
3	Neither distrust nor trust
4	Partially trust
5	Completely trust
6	SPONTANEOUS: Not applicable / No Opinion

Q11126 - TrustGenet: Single coded

In general, would you say you distrust or trust geneticists? *READ OUT*

1	Completely distrust
2	Partially distrust
3	Neither distrust nor trust
4	Partially trust
5	Completely trust
6	SPONTANEOUS: Not applicable / No Opinion

Q11127 - TrustGeol: Single coded

In general, would you say you distrust or trust geologists? *READ OUT*

1	Completely distrust

2	Partially distrust
3	Neither distrust nor trust
4	Partially trust
5	Completely trust
6	SPONTANEOUS: Not applicable / No Opinion

**Q11128 - PandTrustSci:**	**Single coded**

Would you say you now trust scientists more, less, or about the same as you did at the start of the pandemic?
READ OUT

1	Trust them much more
2	Trust them a little more
3	About the same
4	Trust them a little less
5	Trust them much less
999	Don't know **Fixed *Exclusive*

**Q11129 - PandTrustGenet:**	**Single coded**

Would you say you now trust geneticists more, less, or about the same as you did at the start of the pandemic?
READ OUT

1	Trust them much more
2	Trust them a little more
3	About the same
4	Trust them a little less
5	Trust them much less
999	Don't know **Fixed *Exclusive*

**Q11130 - PandTrustGeol:**	**Single coded**

Would you say you now trust geologists more, less, or about the same as you did at the start of the pandemic?
READ OUT

1	Trust them much more

2	Trust them a little more
3	About the same
4	Trust them a little less
5	Trust them much less
999	Don't know **Fixed *Exclusive*

Q11131 - Pfiz: **Single coded**

Would you say you now trust pharmaceutical companies, e.g. Pfizer, more, less or about the same as you did at the start of the pandemic? *READ OUT*

1	Trust them much more 2<br>Trust them a little more
3	About the same
4	Trust them a little less
5	Trust them much less
999	Don't know **Fixed *Exclusive*

Q11132 - Glaxo: **Single coded**

Would you say you now trust pharmaceutical companies, e.g. GlaxoSmithKline, more, less or about the same as you did at the start of the pandemic? *READ OUT*

1	Trust them much more
2	Trust them a little more
3	About the same
4	Trust them a little less
5	Trust them much less
999	Don't know **Fixed *Exclusive*

Q11133 - HadCov: **Single coded**

Do you think that you have, or have had, COVID-19? *READ OUT*

1	Yes, confirmed by a positive test
2	Yes, suspected by a healthcare professional but not tested
3	Yes, my own suspicions
4	No
997	Prefer not to answer **Fixed *Exclusive*

**Q11134 - CovVac:**	**Single coded**

If you were offered a COVID-19 vaccine would you take it? *READ OUT*

1	Yes, and I have already been vaccinated
2	Yes, but I am yet to be vaccinated
3	No, I would not get vaccinated
997	Prefer not to answer **Fixed *Exclusive*
