# Supplementary material for: People with more extreme attitudes towards science have self-confidence in their understanding of science, even if this is not justified
Source: PLoS Biol. 2023 Jan 24;21(1):e3001915. doi: 10.1371/journal.pbio.3001915 (PMC10045565; doi:10.1371/journal.pbio.3001915)
Supplement: S2 Materials — All scripts and data are available at doi: 10.5281/zenodo.7289133. (PDF) [file pbio.3001915.s005.pdf]

Thinking of the stories about science you see or hear in the news, which of the statements on this card would you say best describes you? Please just read out the letter that applies.<br>1 I usually understand what they are talking about<br>2 I sometimes understand what they are talking about<br>3 I usually do not understand what they are talking about<br>4 (SPONTANEOUS: I don't see or hear science news stories)	Wellcome Trust Monitor wave 3	http://doc.ukdataservice.ac.uk/doc/7927/mrdoc/pdf/7927_wtm_w3_appendix_d.pdf
How well informed do you feel, if at all, about science, and scientific research and developments?<br>1. Very well informed<br>2. Fairly well informed<br>3. Not very well informed<br>4. Not at all informed<br>5. (SP; allow DK)	Public Attitudes to Science	https://www.kantar.com/uk-public-attitudes-to-science<br><br>And go to 'download technical report'
I'd now like to ask you about your understanding of different scientific terms that are used in news stories dealing with medical research.<br>First, when you hear the term DNA, how would you rate your understanding of what the term means.<br>1 Very good<br>2 Good<br>3 Some understanding<br>4 Have heard the term but have little understanding of what it means<br>5 Have not heard the term [If answered this, not ask the next DNA question]	Wellcome Trust Monitor wave 2	http://doc.ukdataservice.ac.uk/doc/7315/mrdoc/pdf/7315_questionnaire_and_showcards.pdf

Next, when you hear the term GM or genetically modified, how would you rate your understanding of what the term means?<br>1 Very good<br>2 Good	Wellcome Trust Monitor wave 2	http://doc.ukdataservice.ac.uk/doc/7315/mrdoc/pdf/7315_questionnaire_and_showcards.pdf
3 Some understanding<br>4 Have heard the term but have little understanding of what it means<br>5 Have not heard the term [If answered thi ask the next GM question] ;, not		
Next, when you hear the term natural tion, selec how would you rate your what understanding of the term means.<br>1 Very good<br>2 Good<br>3 Some understanding<br>4 Have heard the term but have little understanding of what it means<br>5 Have not heard the term [If ;, not answered thi ask the next natural selection question]	New question	Based on previous questions, only thing changed was the ‘term’ we are asking for<br><br>http://doc.ukdataservice.ac.uk/doc/7315/mrdoc/pdf/7315_questionnaire_and_showcards.pdf
Next, when you hear the term PCR, how w ›uld you rate your understanding of what the term neans.<br>1 Very good<br>2 Good<br>3 Some understanding<br>4 Have heard the term but have little understanding of what it means<br>5 Have not heard the term [If ;, not answered thi ask the next PCR question]	New question	Based on previous questions, only thing changed was the ‘term’ we are asking for<br><br>http://doc.ukdataservice.ac.uk/doc/7315/mrdoc/pdf/7315_questionnaire_and_showcards.pdf
[Only ask if answered 1-4 on DNA question]	New question	

Now, thinking about the news and stories y<br>heard since the start of the pandemic, do y ou have<br>that your understanding of DNA has… ou feel<br>1. Increased<br>2. Stayed the same<br>3. Decreased<br>Don’t know instead NOT SURE (?)		
[Only ask if answered 1-4 on GM question]	New question	

Now, thinking about the news and stories you have heard since the start of the pandemic, do you feel that your understanding of GM has…<br>1. Increased<br>2. Stayed the same<br>3. Decreased<br>Don’t know instead NOT SURE (?)		
[Only ask if answered 1-4 on natural selection question]<br><br>/, thinking about the news and storie<br>heard nce the start of the pandemic, do yo<br>that understanding of natural selection ıas…<br>1. ıcreased<br>2. tayed the same<br>3. ecreased instead NOT<br>Don’t kno SURE (?)	New question	
[Only ask if answered 1-4 on PCR question]	New question	

Now, thinking about the news and stories y<br>have heard since the start of the pandemic,<br>that your understanding of PCR has... feel<br>1. Increased<br>2. Stayed the same<br>3. Decreased<br>Don't know instead NOT SURE (?)		
And now, thinking back to before the start of the pandemic, to what extent would you have agreed or disagreed with the following statements<br><br>1 - Many claims about the benefits of modern genetic science are greatly exaggerated.<br>2 - Those in charge of new developments in genetic science cannot be trusted to act in society's interests.	Public Attitudes to Genomics: Vignette Studies	This question was initially 3 different questions on this survey. We are happy with either just turning it into one, or have 3 separate questions.<br><br>http://doc.ukdataservice.ac.uk/doc/5444/mrdoc/pdf/5444userguide.pdf<br><br>https://beta.ukdataservice.ac.uk/datacatalogue/studies/study?id=5444#!/details

3 - On balance, the advantages of genetically modified (GM) foods outweigh any dangers.<br><br>• Strongly agree<br>• Agree<br>• Neither agree or disagree<br>• Disagree<br>• Strongly disagree<br>How do you think your view has changed in the last year		

"For each of the following statements, please say whether you think it is definitely true, probably true, probably false or definitely false. If you don't know, just say so and we'll go on to the next one:"<br><br>All plants and animals have DNA (TRUE)<br>The oxygen we breathe comes from plants (TRUE)<br>The cloning of living things produces genetically identical copies (TRUE)<br>By eating a genetically modified fruit, a person's genes could also become modified (FALSE)<br>All radioactivity is human-made (FALSE)<br>It is the mother's genes that determine the sex of the child (FALSE)<br>Electrons are smaller than atoms (TRUE)<br>Tomatoes do not naturally contain genes; genes are only found in genetically modified tomatoes (FALSE)<br>Dinosaurs and humans share a common ancestor [TRUE]<br>The spread of new variants of viruses can occur through natural selection [TRUE] Covid 19 is caused by bacteria [FALSE]	Wellcome Trust Monitors, Public Understanding of Genomics and the Dynamics of Opinion Change: a Panel Study and new questions	http://doc.ukdataservice.ac.uk/doc/7927/mrdoc/pdf/7927_wtm_w3_appendix_d.pdf<br><br>All plants and animals have DNA (TRUE)<br>The oxygen we breathe comes from plants (TRUE)<br>The cloning of living things produces genetically identical copies<br>By eating a genetically modified fruit, a person's genes could also become modified<br><br>All radioactivity is human-made (FALSE) – original wording is 'All radioactivity is man made' we change it to be inclusive<br><br>It is the mother's genes that determine the sex of the child<br>Electrons are smaller than atoms<br><br>https://beta.ukdataservice.ac.uk/datacatalogue/studies/study?id=5147#!/documentation<br>http://doc.ukdataservice.ac.uk/doc/5147/mrdoc/pdf/5147userguide.pdf<br><br>Tomatoes do not naturally contain genes; genes are only found in genetically modified tomatoes<br><br>Questions we made up:<br><br>Dinosaurs and humans share a common ancestor [TRUE]<br>The spread of new variants of viruses can occur through natural selection [TRUE] Covid 19 is caused by a bacteria [FALSE]

Viruses are smaller than bacteria [TRUE]		Viruses are smaller than bacteria [TRUE]
How optimistic are you about the possibility of improved healthcare as a result of genetic research?<br>• Very optimistic,<br>• Somewhat optimistic,	Wellcome Trust Monitor wave 2	http://doc.ukdataservice.ac.uk/doc/7315/mrdoc/pdf/7315_questionnaire_and_showcards.pdf

• Not too optimistic, OR, Not at all optimistic		
Which of these statements best describes your relationship with science?<br>A. I feel connected with science – I actively seek out science news, events, activities or entertainment B. I'm interested in science, but I don't make a special effort to keep informed C. Science is not for me<br>Might be part of access section	Public Attitudes to Science	https://www.kantar.com/uk-public-attitudes-to-science<br><br>Go to 'Download technical report'
On balance, which of the following best describes your attitude to the scientific study of genetics at present? [we prioritise this question, we rather know the true answer to genetics]<br>• Very positive<br>• Slightly positive<br>• Undecided<br>• Slightly negative<br>• Very negative<br>I don't know enough to form a judgement	Genomics England polling	https://www.ipsos.com/sites/default/files/ct/publication/documents/201904/genomics_england_polling_report_public.pdf<br><br>This was altered from genomics (in the original survey) to genetics

Which, if any, of these have you seen, read or heard about over the last three months?<br>• Services offering online genetic profiling for individuals, such as “23andMe” or “AncestryDNA”<br>• New techniques for scientists to be able to edit the genomes of plants, animals or humans<br>• PCR testing for Covid-19	Genomics England polling	https://www.ipsos.com/sites/default/files/ct/publication/documents/201904/genomics_england_polling_report_public.pdf<br><br>Answer options were changed from original

• Natural selection operating on viruses<br>• Any negative or concerning stories about genomic research or medicine • None of the above<br>Might be part of access section		

<table>
<tr>
<td>Who would you trust to provide accurate and reliable information about COVID-19?
[Put instructions on how to answer the question]
<table>
<tr><th>People and organisations</th><th>Covid-19</th></tr>
<tr><td>Work colleagues</td><td></td></tr>
<tr><td>The government</td><td></td></tr>
<tr><td>The government’s scientific advisers</td><td></td></tr>
<tr><td>Celebrities and public figures</td><td></td></tr>
<tr><td>Non-for-profit organisations/charities</td><td></td></tr>
<tr><td>NHS spokesperson</td><td></td></tr>
<tr><td>Research Scientist / Universities</td><td></td></tr>
<tr><td>Family / friends</td><td></td></tr>
<tr><td></td><td></td></tr>
<tr><td></td><td></td></tr>
</table>
</td>
<td></td>
<td>A version of this question was provided by you (Richard) so not sure where it is from. We have altered it significantly.</td>
</tr>
</table>

Which media outlets would you trust to provide accurate and reliable information about COVID-19?
[Put instructions on how to answer the question]

**Media**	**Covid19**
Government website	
TV - News	
Topical TV shows (e.g. 'The One show' or 'Have I got news for you')	
Celebrities/Influencers on social media eg Facebook, Twitter	
Organisations eg charities/universities/professional bodies on Social media	
Individual scientists on Social media	
Other individuals on Social media	
YouTube	
Websites which focus on this topic	
Newspapers	
Online-only news sites e.g. Huffington Post, Google News	
Online news sites of traditional media (BBC news etc)	
Radio – BBC National (e.g. BBC Radio1)	
Radio – BBC local (e.g. BBC Wiltshire)	
Radio – Commercial radio stations (e.g. Capital FM)	

Over the last few months, how much, if anything, have you heard or read about issues to do with genes and genetics?<br>• A great deal<br>• Quite a lot	Public Attitudes to Genomics: Vignette Studies	http://doc.ukdataservice.ac.uk/doc/5444/mrdoc/pdf/5444userguide.pdf<br>https://beta.ukdataservice.ac.uk/datacatalogue/studies/study?id=5444#!/details
• A small amount<br>• Not very much<br>• Not at all		
Over the past few months, how much, if at all, have you thought about issues to do with genes and genetics? • A great deal<br>• Quite a lot<br>• A small amount<br>• Not very much • Not at all.	Public Attitudes to Genomics: Vignette Studies	http://doc.ukdataservice.ac.uk/doc/5444/mrdoc/pdf/5444userguide.pdf<br>https://beta.ukdataservice.ac.uk/datacatalogue/studies/study?id=5444#!/details
Which of the following statements on this card do you most agree with? These days I hear and see …<br>1. … far too much information about science<br>2. … too much information about science<br>3. … the right amount of information about science<br>4. … too little information about science<br>5. … far too little information about science	Public Attitudes to Science	https://www.kantar.com/uk-public-attitudes-to-science
In general, would you say you distrust or trust scientists:<br>• Completely distrust<br>• Partially distrust<br>• Neither distrust nor trust<br>• Partially trust<br>• Completely trust<br>Not applicable / No Opinion		

In general, would you say you distrust or trust geneticists:<br>• Completely distrust<br>• Partially distrust<br>• Neither distrust nor trust<br>• Partially trust<br>• Completely trust<br>Not applicable / No Opinion		

In general, would you say you distrust or trust geologists:<br>• Completely distrust<br>• Partially distrust<br>• Neither distrust nor trust<br>• Partially trust<br>• Completely trust<br>Not applicable / No Opinion		
Would you say you personally trust scientists more, less, or about the same as you did since the start of the pandemic?<br><br>Trust them much more<br>Trust them a little more<br>About the same<br>Trust them a little less<br>Trust them much less<br>Don’t know	New question	

Would you say you personally trust geneticists more, less, or about the same as you did since the start of the pandemic?<br><br>Trust them much more<br>Trust them a little more<br>About the same<br>Trust them a little less<br>Trust them much less<br>Don’t know	New question	
Would you say you personally trust geologists more, less, or about the same as you did since the start of the pandemic ?<br><br>Trust them much more<br>Trust them a little more<br>About the same<br>Trust them a little less<br>Trust them much less Don’t know	New question	

Experiment<br><br>Ask half the sample:<br><br>Would you say you personally trust pharmaceutical companies, eg Pfizer, more, less or about the same as you did since the start of the pandemic?<br><br>Trust them much more<br>Trust them a little more<br>About the same<br>Trust them a little less<br>Trust them much less<br>Don't know<br><br>Ask the other half of the sample:<br><br>Would you say you personally trust pharmaceutical companies, eg GlaxoSmithKline, more, less or about the same as you did since the start of the pandemic?<br><br>Trust them much more<br>Trust them a little more<br>About the same<br>Trust them a little less<br>Trust them much less<br>Don't know	New question	
Do you think that you have, or have had, COVID19?		Covid-19 Self-report core survey<br>https://ihccglobal.org/covid-19-survey-downloads/

Yes, confirmed by a positive test<br>Yes, suspected by a healthcare professional but not tested<br>Yes, my own suspicions<br>No<br>Don’t want to answer [this was added by GenSoc]		
If you were offered a COVID-19 vaccine would you take it?<br><br>Yes, and I have already been vaccinated<br>Yes, but I am yet to be vaccinated<br>No, I would not get vaccinated<br>Prefer not to answer	New question	
