# Supplementary material for: People with more extreme attitudes towards science have self-confidence in their understanding of science, even if this is not justified
Source: PLoS Biol. 2023 Jan 24;21(1):e3001915. doi: 10.1371/journal.pbio.3001915 (PMC10045565; doi:10.1371/journal.pbio.3001915)
Supplement: S2 Table — All scripts and data are available at doi: 10.5281/zenodo.7289133. (PDF) [file pbio.3001915.s007.pdf]

**A. Tukey post hoc tests: Self assessment versus vaccine classes**	Estimate	Std. Error	t value	Pr(> I tI)
Prefer not to answer - No, I would not get vaccinated	-0.065	0.032	-1.997	0.17
Yes, and I have already been vaccinated - No, I would not get vaccinated	0.002	0.019	0.105	1
Yes, but I am yet to be vaccinated - No, I would not get vaccinated	0.025	0.021	1.194	0.603
Yes, and I have already been vaccinated - Prefer not to answer	0.067	0.027	2.493	0.053
Yes, but I am yet to be vaccinated - Prefer not to answer	0.09	0.028	3.204	**0.006**
Yes, but I am yet to be vaccinated - Yes, and I have already been vaccinated	0.023	0.01	2.232	0.102
**B. Tukey post hoc tests: Scientific knowledge versus vaccine classes**				
Prefer not to answer - No, I would not get vaccinated	-0.025	0.051	-0.486	0.958
Yes, and I have already been vaccinated - No, I would not get vaccinated	0.12	0.031	3.908	**0.0005**
Yes, but I am yet to be vaccinated - No, I would not get vaccinated	0.134	0.034	4.001	**0.0003**
Yes, and I have already been vaccinated - Prefer not to answer	0.145	0.042	3.438	**0.003**
Yes, but I am yet to be vaccinated - Prefer not to answer	0.159	0.044	3.592	**0.002**
Yes, but I am yet to be vaccinated - Yes, and I have already been vaccinated	0.014	0.017	0.838	0.82
**C. Tukey post hoc tests: OSD versus vaccine classes**				
Prefer not to answer - No, I would not get vaccinated	0.039	0.047	0.831	0.824
Yes, and I have already been vaccinated - No, I would not get vaccinated	0.118	0.028	4.257	**0.0001**
Yes, but I am yet to be vaccinated - No, I would not get vaccinated	0.109	0.03	3.603	**0.002**
Yes, and I have already been vaccinated - Prefer not to answer	0.08	0.039	2.06	0.149
Yes, but I am yet to be vaccinated - Prefer not to answer	0.07	0.04	1.738	0.278
Yes, but I am yet to be vaccinated - Yes, and I have already been vaccinated	-0.009	0.015	-0.621	0.917
