# Supplementary material for: People with more extreme attitudes towards science have self-confidence in their understanding of science, even if this is not justified
Source: PLoS Biol. 2023 Jan 24;21(1):e3001915. doi: 10.1371/journal.pbio.3001915 (PMC10045565; doi:10.1371/journal.pbio.3001915)
Supplement: S3 Table — For Trust, Hype, and GM, these are class −2. For vaccine, it is those who will not have it. All scripts and data are available at doi: 10.5281/zenodo.7289133. (PDF) [file pbio.3001915.s008.pdf]

Variable	Dip	P	N	Median	Prop_sig
Trust	0.0718	0.05330	47	0.0759	0.106
Hype	0.0517	0.81700	29	0.0862	0.247
GM	0.0667	0.00109	105	0.0862	0.134
Vaccine	0.0471	0.17700	85	0.0690	0.046
