# Supplementary material for: People with more extreme attitudes towards science have self-confidence in their understanding of science, even if this is not justified
Source: PLoS Biol. 2023 Jan 24;21(1):e3001915. doi: 10.1371/journal.pbio.3001915 (PMC10045565; doi:10.1371/journal.pbio.3001915)
Supplement: S4 Table — A high correlation implies those who answered 1 question correctly[incorrectly] also answered the other one correctly[incorrectly]. All scripts and data are available at doi: 10.5281/zenodo.7289133. (PDF) [file pbio.3001915.s009.pdf]

	Q 1	Q 2	Q 3	Q 4	Q 5	Q 6	Q 7	Q 8	Q 9	Q 10	Q 11	Q 12
Q 1		0.11****	0.13****	0.11****	0.07**	0.00	0.07***	0.13****	0.09****	0.09****	0.06**	0.04
Q 2			0.07***	0.02	-0.01	-0.03	0.03	-0.01	0.04	0.01	-0.01	0.03
Q 3				0.16****	0.16****	0.07**	0.17****	0.17****	0.06*	0.07***	0.19****	0.15****
Q 4					0.30****	0.20****	0.11****	0.31****	0.07**	0.07**	0.24****	0.10****
Q 5						0.21****	0.19****	0.38****	0.06*	0.16****	0.33****	0.17****
Q 6							-0.01	0.20****	-0.04	0.03	0.20****	0.01
Q 7								0.20****	0.13****	0.21****	0.21****	0.28****
Q 8									0.09****	0.17****	0.30****	0.17****
Q 9										0.18****	0.03	0.08***
Q 10											0.08***	0.19****
Q 11												0.19****
Q 12												

"
