# Supplementary material for: People with more extreme attitudes towards science have self-confidence in their understanding of science, even if this is not justified
Source: PLoS Biol. 2023 Jan 24;21(1):e3001915. doi: 10.1371/journal.pbio.3001915 (PMC10045565; doi:10.1371/journal.pbio.3001915)
Supplement: S1 Results — All scripts and data are available at doi: 10.5281/zenodo.7289133. (PDF) [file pbio.3001915.s010.pdf]

1. Supplementary results 1: the data accords with expectations of unpolarised science.

If results as seen for politicized attitudes [1] hold in the current context, we expect to see at both extremes of attitudinal position high scores in the objective knowledge tests. Conversely, if adequately depolarised, we may expect a positive correlation (without allowance for covariates). We observe that for both Trust and Hype, the higher the acceptance the higher the scientific knowledge. For both the correlation is moderate (Fig SR1: Spearman rank tests: Trust, rho=0.204, P=2 x 10^{-16}; Hype rho=0.28, P<2 x 10^{-16}).

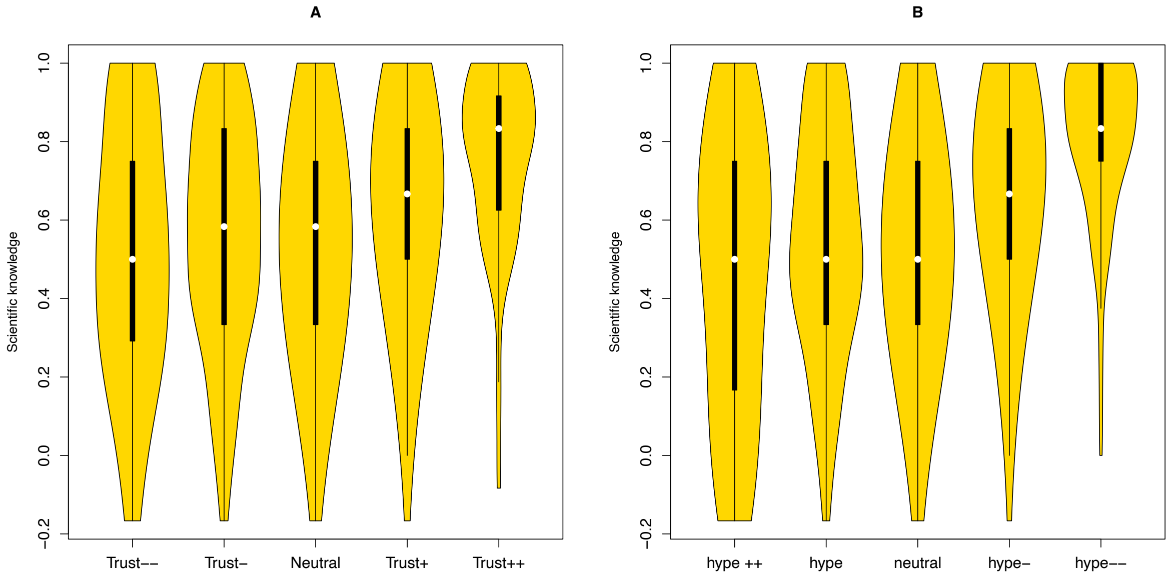

Fig.SR1 Objective science knowledge as a function of attitudinal position A). Trust and B). Hype. For both plots the more rejectionist stance is plotted to the left. Both are highly significantly tending to higher objective knowledge scores when attitudinal position is more accepting (Spearman rank tests: trust, rho=0.204, P=2 x 10^{-16}; hype rho=0.28, P<2 x 10^{-16}).

Polarised science tends to reveal as high educational levels at extremities of attitudinal position [1]. Respondents were classified as to whether they have a degree level qualification, qualifications below degree level or no qualifications (scored as 2, 1 and 0). We find that for both attitudinal positions, those with no qualifications are enriched in the grouping with the most negative attitudes (Fig SR2). Conversely, those more accepting (Trust ++, Hype --) are greatly enriched for those with University level degrees. Overall, there is a positive correlation between both trust/hype and

educational level (Spearman rank test: Trust: rho=0.11, $P=6.9 \times 10^{-7}$; Hype: rho=0.15, $P=2.6 \times 10^{-12}$).

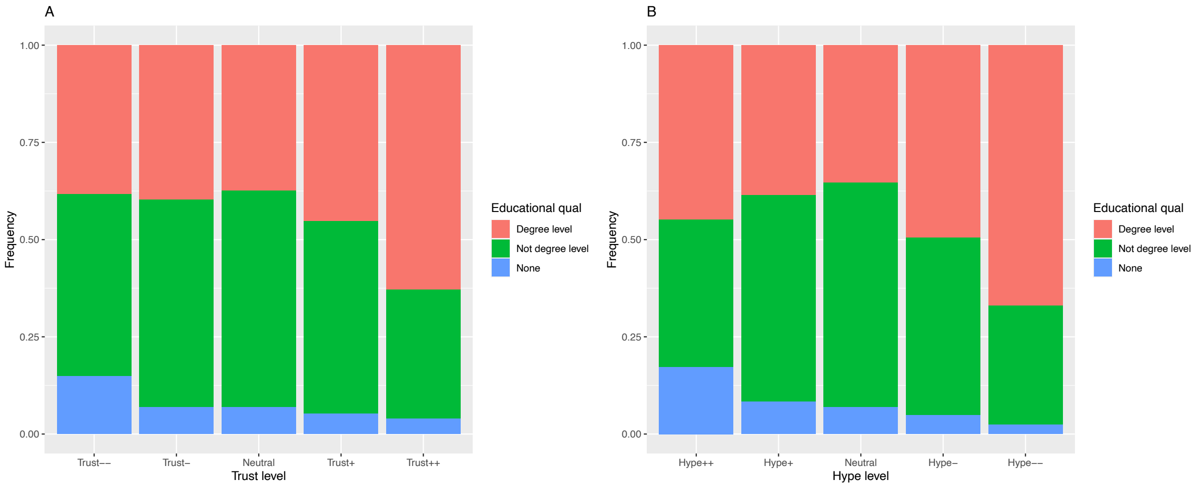

Fig.SR2 Educational attainment as a function of attitudinal position A Trust, B. Hype Both are plotted with more rejectionist views to the left (Trust --, Hype ++).

If polarization were present we should expect that for both parameters (hype and trust) there would be death of individuals with neutral stances and hence a U or M shaped distribution relating frequency to attitudinal position [2]. This we do not observe (Fig SR3). As the distributions are profoundly n shaped we provide no statistics here as the trend is self-evident. We are unable to address whether attitudinal position is predicted by political position.

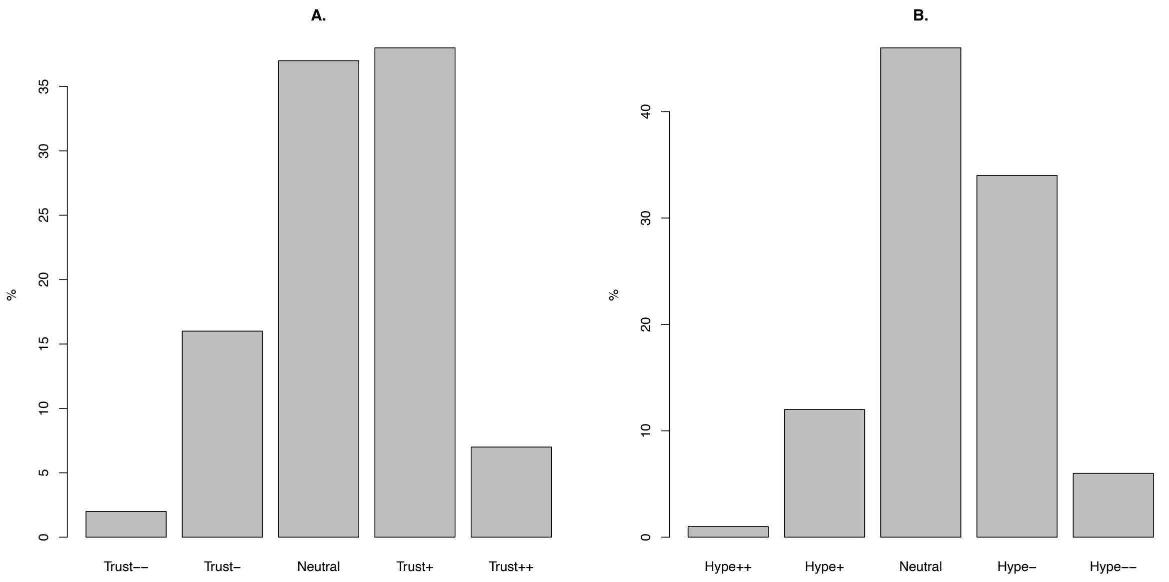

Fig SR3. Frequency by attitudinal position for A) Trust and B) Hype. In both cases more rejectionist attitudes are towards the left.

We can also examine an effect of political identity. Here the analysis is not so clear. For the Trust parameter those at the two extremes are more left wing than those in moderate positions, arguing against political polarization. For Hype by contrast, there is a trend for more accepting/positive attitudes to be associated with more left wing identity. Nonetheless, the most rejectionist class scores as politically neutral on the average, rendering any claim that these are right wing ideologs implausible (Note conservative voters do score highly positive in this metric (S3 Fig).

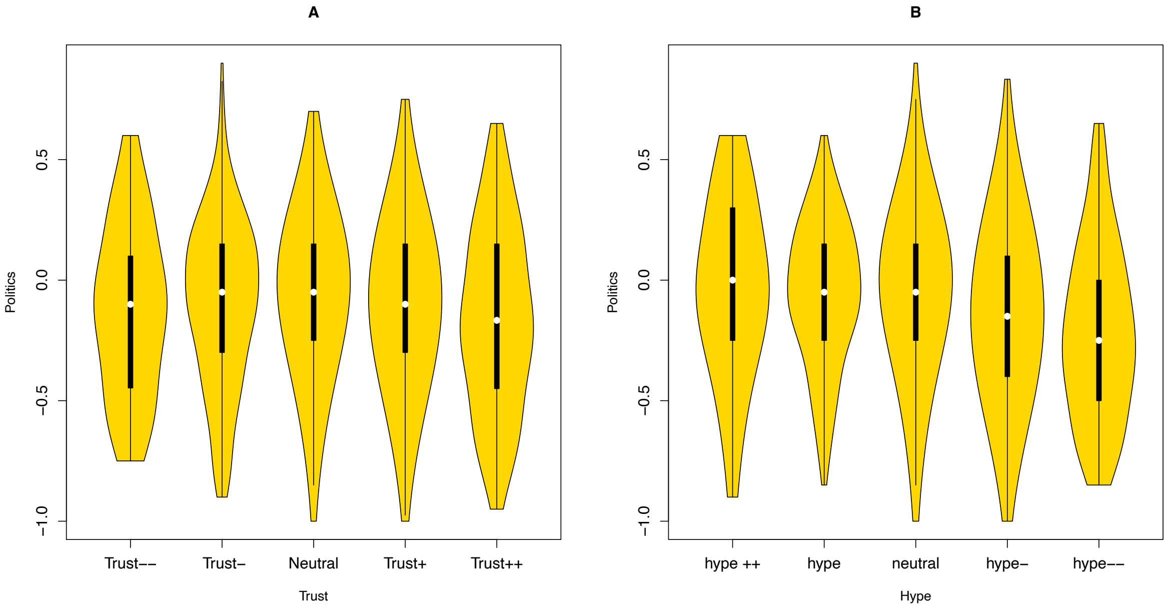

Fig SR4. Political score for A) Trust and B) Hype. In both cases more rejectionist attitudes are towards the left. The more right wing the attitudes the more positive the politics score.

1. Drummond C, Fischhoff B. Individuals with greater science literacy and education have more polarized beliefs on controversial science topics. Proc Natl Acad Sci U S A. 2017;114(36):9587-92. Epub 20170821. doi: 10.1073/pnas.1704882114.
2. Bramson A, Grim P, Singer DJ, Berger WJ, Sack G, Fisher S, et al. Understanding polarization: Meanings, measures, and model evaluation. Philos Sci. 2017;84(1):115-59. doi: Doi 10.1086/688938.
