# Supplementary material for: People with more extreme attitudes towards science have self-confidence in their understanding of science, even if this is not justified
Source: PLoS Biol. 2023 Jan 24;21(1):e3001915. doi: 10.1371/journal.pbio.3001915 (PMC10045565; doi:10.1371/journal.pbio.3001915)
Supplement: S2 Results — All scripts and data are available at doi: 10.5281/zenodo.7289133. In this instance, consult zip archive S_results_2.zip. (PDF) [file pbio.3001915.s011.pdf]

Supplementary results 2: Repeat of main results for modified input data

A concern with our main analysis is that we provide analysis of both biological/genetical questions and more general science related questions. Here we reanalyse the data but this time defining subjective understanding by reference to the four questions (q 3-6) that were explicitly genetical (see methods below). Similarly, we define scientific knowledge as explicitly only biological genetical knowledge, i.e. removing questions about radioactivity and atoms. Qualitatively different means a switch between P <0.05 and P>0.05 in either direction. All figures and tables are updated. All scripts and data are available at doi: 10.5281/zenodo.7289133. The results that have changed are shown in red font. Supplementary figures referred to are not presented but can be derived by running the scripts at doi: 10.5281/zenodo.7289133. What follows is the same text as the main paper.

PART I: ATTITUDINAL STRENGTH IS PREDICTED BY SUBJECTIVE UNDERSTANDING

Prediction 1a. Attitude strength correlates with subjective understanding controlling for covariates: Attitude strength is positively correlated with subjective understanding (Spearman rank test: Trust: rho=0.23, P=2 x 10^{-16}; Hype, rho=0.28, P=2 x 10^{-16}). This is robust to covariate control (Table 1: Trust, partial rho = 0.216, P=5 x 10^{-23}; Hype partial rho = 0.25, P=8 x 10^{-31}). The attitude strength scores are otherwise only predicted by educational attainment with higher attainment associated with stronger attitudes (Table 1). We conclude that stronger attitudes are associated with stronger subjective assessment of understanding.

Table 1 Correlation and partial correlation analysis of between a) modular Trust score b) modular Hype score and subjective science understanding (SAU) controlling for age, religiosity, political identity, and educational attainment. The values above the diagonal are the spearman rho values for each comparison. Those below the diagonal are the same pairwise partial correlations controlling for all other variables. All values in bold are significant (at under 0.05). All non-significants are P>0.05. N=2051.

a.

	Education	Age	Religiosity	Politics	SAU	|Trust|
**Education**	-	**-0.14**	0.04	**-0.26**	**0.25**	**0.10**
**Age**	**-0.08**	-	**0.20**	**0.24**	**-0.15**	-0.01
**Religiosity**	**0.10**	**0.17**	-	**0.17**	-0.04	0.00
**Politics**	**-0.22**	**0.18**	**0.15**	-	**-0.17**	**-0.08**
**SAU**	**0.19**	**-0.1**	-0.02	-0.08	-	**0.23**
**|Trust|**	0.04	0.04	0.01	**-0.05**	**0.21**	-

b.

	Education	Age	Religiosity	Politics	SAU	|Hype|
**Education**	-	**-0.14**	0.04	**-0.26**	**0.25**	**0.15**
**Age**	**-0.08**	-	**0.20**	**0.24**	**-0.15**	-0.01
**Religiosity**	**0.10**	**0.17**	-	**0.17**	-0.04	0.00
**Politics**	**-0.21**	**0.18**	**0.15**	-	**-0.17**	**-0.14**
**SAU**	**0.218**	**-0.11**	-0.02	**-0.07**	-	**0.27**
**|Hype|**	**0.07**	**0.05**	0.01	**-0.09**	**0.24**	-

Predictions 1b/1c: Extreme negative and positive attitudes towards genetics are associated with subjective understanding: The model requires that attitude strength increases

with subjective understanding for both those with negative and positive attitudes. In accord with this we observe a U-shaped distribution between subjective understanding and both Trust and Hype (Fig 1). As predicted by the model (prediction 1b), a quadratic fit is highly significantly better than a linear fit in both instances: Trust: adjusted R^2 for quadratic model = 0.057; for linear model = 0.020; Hype: adjusted R^2=0.075 for quadratic model, for linear model= 0.029; $P<10^{-16}$ in both cases for significance of difference between quadratic and linear fit.

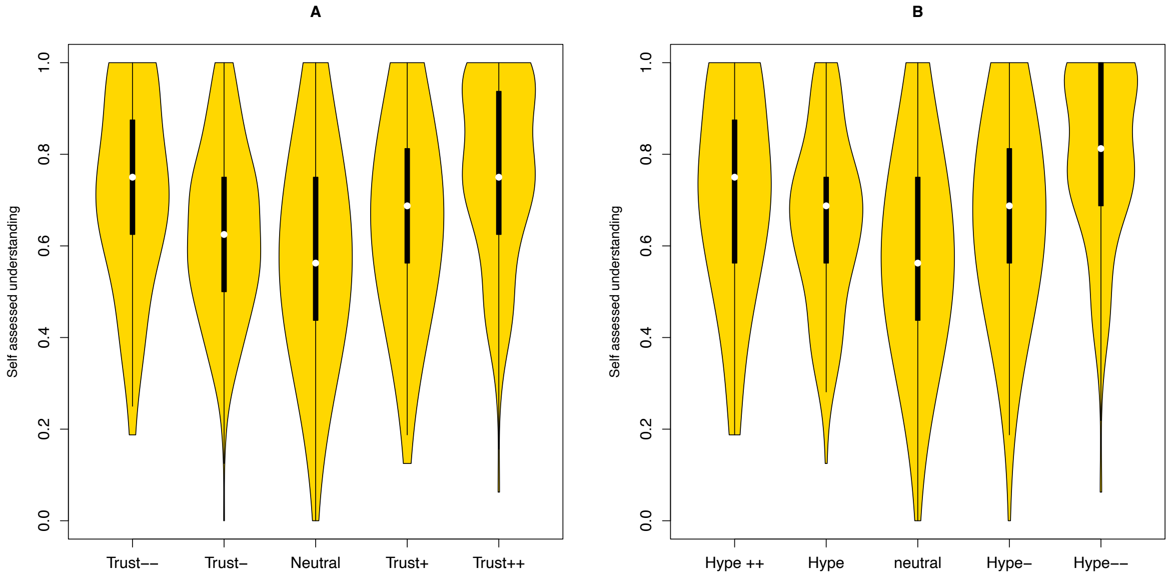

Fig.1 Subjective science understanding as a function of attitudinal position for A) Trust B. Hype. Both are plotted with more rejectionist views to the left (Trust --, Hype ++). Scoring the x values, -2 to +2, a quadratic fit is highly significant in both instances ($P<10^{-16}$ in both cases).

Given that there is a relatively low number of individuals in the class considered most negative (N=47 for Trust, N=29 for Hype), it is possible that the U-shaped function is an artefact of skewed sampling with larger classes disproportionately influencing the form of the curve. To address this we resample the data, this time allowing equal numbers in each classification (-2, -1, 0 etc), this number being the size of the smallest of the classifications (in these two incidences this is the most negative class). Thus, if there are 47 in the least trusting class, we resample without replacement 47 from each of the other four classes generating a balanced sample 47 x 5 = 235 long. We then repeat the test for linear and quadratic fits and ask in what proportion of resamplings is a quadratic fit significantly better than the linear fit at $P<0.05$. Repeating for 10,000 randomizations we find for Trust that 99.95% of randomizations have an improved quadratic fit and for Hype 98.6% have an improved quadratic fit. We conclude that the observed improved quadratic fit is not an artefact of skewed samples sizes.

Given this improvement of the quadratic fit, we additionally consider the correlation between subjective understanding and attitude score for both halves of the distribution separately (Prediction 1c). This confirms the trends (Trust >= 0, rho = 0.26, P=2 x 10^{-16}; Trust <= 0, rho = -0.132, P=8.5 x 10^{-6}; Hype >=0, rho = 0.29, P = 2 x 10^{-16}; Hype <=0, rho =-0.16, P=2.5 x 10^{-8}). We conclude that as subjective understanding increases so too does attitudinal extremity, in both positive and negative directions.

PART II OBJECTIVE-SUBJECTIVE DEFICIT AS A PREDICTOR OF NEGATIVE ATTITUDES

Part IIa

Prediction 2a: An excess with low knowledge but high subjective understanding: The above establishes a general attitude strength-subjective understanding effect but establishes nothing about valence (i.e. directionality of attitude). If there exists a subpool of individuals with high subjective understanding but low knowledge we expect a quadratic fit between the two parameters. Overall, we find a moderate correlation between subjective and objective measures (Fig 2: rho = 0.40, P< 2 x 10^{-16}). This implies that, to a first approximation, the UK public have an accurate perception of their own scientific abilities. The quadratic fit is, however, a significantly better fit than a linear fit (linear model: Adjusted R^2=0.159; quadratic model: Adjusted R^2=0.163, the quadratic being the significantly better fit, P=0.00056). This is consistent with a subpopulation at low levels of scientific knowledge with higher subjective understanding scores than expected under a simple linear model, potentially compatible with a relatively rare low knowledge but high self-confidence subpopulation.

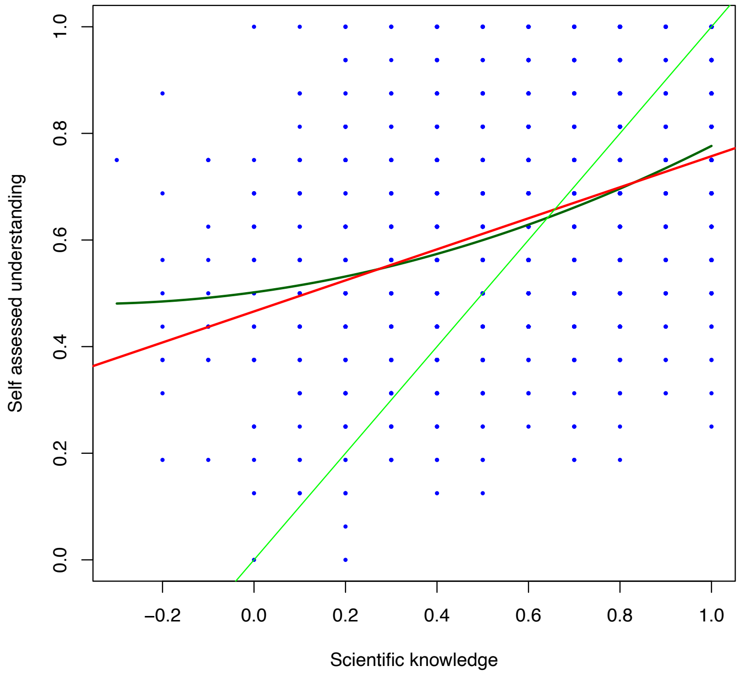

Fig 2. Subjective science understanding and objective science knowledge covary. The linear fit is shown in red, quadratic in dark green. The light green line is the line of slope 1 to define OSD.

Prediction 2b: The subjective-objective deficit is predicted by negativity of attitude, higher religiosity and lower educational attainment: For both Trust and Hype, the higher the acceptance, the higher the scientific knowledge (Spearman rank tests: Trust, rho=0.201, P=2 x 10^{-16}; Hype rho=0.28, P<2 x 10^{-16}) (Fig S1). Given this and the U-shaped function in prediction 1b/1c, we might expect a subjective-objective deficit (OSD) that covaries with attitudinal position. A negative score implies over confidence i.e. a deficit in scientific knowledge below subjective levels. As previously reported [1-3], most people are over-confident having a negative OSD (see fig 2).

Given prior trends (Fig S1, Fig 1), OSD should become more negative as attitudinal position becomes more negative. This is indeed observed (Figure 3: Trust, rho=0.074 P=0.0007; Hype: rho=0.14 p< 2.9 x 10^{-10}). Heterogeneity with a low large deficit for the strong rejectors is also seen using metric of population relative OSD based on differences in Z scores [4] (ANOVA: Trust: P=0.00012 Hype, P=2.5 x 10^{-7}). We conclude that individuals with a greater deficit (i.e. more negative OSD) are more likely to hold negative attitudes towards genetics.

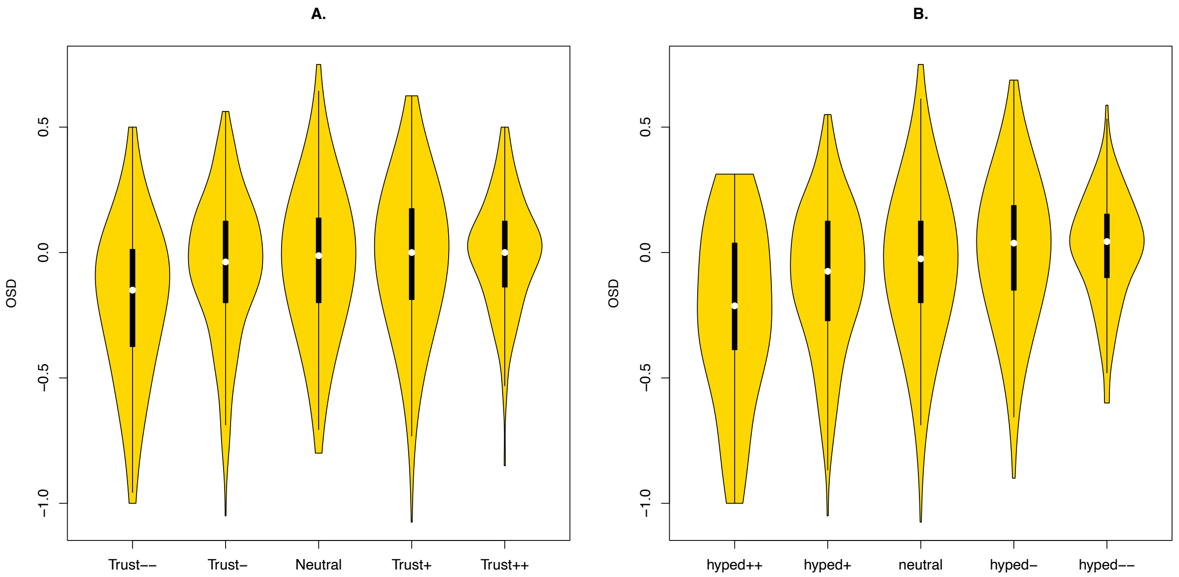

Figure 3. Objective subjective deficit (OSD) as a function of attitude for A. Trust and B. Hype. Both are plotted with more rejectionist views to the left (Trust --, Hype ++).

Prediction 2c: OSD – attitude correlations are robust to covariate control: The OSD-attitude correlations may be explained as a consequence of covariates. We again consider four parameters: age, religiosity, political identity and educational level. The OSD-attitude result is robust to covariate control (Trust v OSD, partial rho = 0.08, P= 1.6 x 10^{-4}; OSD Hype, partial rho = 0.14, P= 3 x 10^{-10} (Tables 2a and 2b). We conclude that more negative attitudes are associated with low objective knowledge compared to subjective knowledge and that this trend is not explained by the covariates age, religiosity, political identity and educational level.

Table 2 Correlation and partial correlation analysis of age, religiosity, educational attainment, political identity, gap and a) Trust and b) Hype scores. The values above the diagonal are the spearman rho values for each comparison. Those below the diagonal are the same pairwise partial correlations controlling for all other variables. All values in bold are significant (at under 0.05). All non-significants are P>0.05. N=2051. [note change in 2b, partial politics OSD now not significant]

a

	Education	Age	Religiosity	Politics	OSD	Trust
**Education**	-	**-0.14**	0.04	**-0.26**	**0.10**	**0.11**
**Age**	**-0.11**	-	**0.20**	**0.24**	**0.09**	-0.03
**Religiosity**	**0.11**	**0.18**	-	**0.17**	**-0.07**	**-0.05**
**Politics**	**-0.23**	**0.19**	**0.14**		**-0.07**	**-0.05**
**OSD**	**0.10**	**0.13**	**-0.08**	**-0.05**		**0.07**
**Trust**	**0.09**	-0.01	-0.04	-0.01	**0.06**	-

b.

	Education	Age	Religiosity	Politics	OSD	Hype
**Education**	-	**-0.14**	0.04	**-0.26**	**0.10**	**0.15**
**Age**	**-0.11**	-	**0.20**	**0.24**	**0.09**	-0.04
**Religiosity**	**0.11**	**0.18**	-	**0.17**	**-0.07**	**-0.07**
**Politics**	**-0.22**	**0.19**	**0.14**	**-**	**-0.07**	**-0.15**
**OSD**	**0.09**	**0.13**	**-0.08**	-0.04	-	**0.14**
**Hype**	**0.12**	0.0	**-0.05**	**-0.10**	**0.12**	-

Independent of the attitudinal positions, we find that a knowledge deficit (over-confidence) is predicted by higher religiosity (rho=-0.07), lower educational attainment (rho=0.1), more right-wing attitudes (rho=-0.07) and lower age (rho=0.09) (Prediction test 2 corollary: Table 3a). These results are robust to the relative OSD metric [4] (Table 3b), excepting for the OSD education correlation.

Table 3 Correlation and partial correlation analysis of age, religiosity, educational attainment, and OSD score. The values above the diagonal are the spearman rho values for each comparison. Those below the diagonal are the same pairwise correlations controlling for all other variables. All values in bold are significant (at under 0.005). All non-significants are P>0.05. N=2051. Table a employs the absolute gap metric while b. employs a Z based metric.

a.

	Education	Age	Religiosity	Politics	OSD
**Education**	-	**-0.14**	0.04	**-0.26**	**0.10**
**Age**	**-0.11**	-	**0.20**	**0.24**	**0.09**
**Religiosity**	**0.11**	**0.18**	-	**0.17**	**-0.07**
**Politics**	**-0.24**	**0.19**	**0.14**	**-**	**-0.07**
**OSD**	**0.11**	**0.13**	**-0.09**	**-0.05**	-

b.

	Education	Age	Religiosity	Politics	OSD
**Education**	-	**-0.14**	0.04	**-0.26**	0.02
**Age**	**-0.10**	-	**0.20**	**0.24**	**0.12**
**Religiosity**	**0.10**	**0.18**	-	**0.17**	**-0.05**
**Politics**	**-0.24**	**0.19**	**0.15**	**-**	-0.01
**OSD**	0.04	**0.14**	**-0.07**	-0.02	-

Considering those cases where the attitudinal score is less than or equal to zero, we find that the slope of the subjective understanding versus attitude score is significantly different for Trust and Hype, (and more negative) than for the objective knowledge (Prediction 2d; Table 4). This accords with the hypothesis that as attitudes become more negative, subjective understanding increases more than does objective knowledge.

Table 4. Test of difference in slope for the subjective and objective knowledge scores as a function of attitude for cases where attitudes score is =< 0.

Attitude	Slope subjective	SEM	Slope objective	SEM	*t*	*P*	*df*
**Trust**	-0.0444	0.0090	-0.0026	0.0150	-2.3866	0.0085	2250

Hype	-0.0609	0.0099	0.0009	0.0163	-3.2462	0.0006	2458

Part IIb

Existing evidence suggests that high OSD predicts negative attitudes for both the GM and the vaccine issues [4, 5]. Here, we ask whether our data replicates both OSD effects. In the case of GM we also investigate attitudinal strength (Prediction group 1) as the structure of the test is comparable to that employed above.

Replication 1, GM attitudes: To address the GM issue, after asking questions 1 (Hype) and 2 (Trust), we asked: "On balance, the advantages of genetically modified (GM) foods outweigh any dangers.". This was scored -2 to +2, with, as before, more negative attitudes being more negative in score. The distribution is approximately normal (Fig S1A). The attitude strength predictions are all upheld: (1a) attitude strength is positively correlated with subjective understanding (rho=0.19, P<2 x 10^{-16}), (1b) a U-shaped function relates subjective understanding and attitudinal position (Fig 4A: quadratic fit, adjusted R^2=0.048; P=2 x 10^{-16}; linear fit adjusted R^2=0.012, P=4.7 x 10^{-7}, P for improved fit= 22 x 10^{-16}) and (1c) subjective understanding is correlated with attitude in the expected manner (GM >=0, rho = 0.25, P=2 x 10^{-16}; GM<= 0, rho = -0.112, P=1.3 x 10^{-5}). In none of 10,000 resamplings, in which all classes are the length of the smallest class (strongly accepting in this case), is the improved quadratic not found.

The OSD/valence predications are also largely upheld. The modular attitude strength verses subjective understanding (rho=0.18, P<2 x 10^{-16}) is a stronger effect that the relationship between objective knowledge and attitudinal position (Spearman rank correlation rho=0.149, P=1.1 x 10^{-11} (Fig 4B) and consequently, OSD is predicted by attitudinal position (rho=0.07, P=0.0009), this being robust to covariate control (partial rho = 0.079, P=3.5 x 10^{-4}) (Table 5). For frequencies see S1A Fig, for educational attainment see S1B Fig for political identify see S1C Fig. However, the one exception is that when we restrict analysis to only instances where the attitude score is less than or equal to zero, the slopes of the two lines are not significantly different, although the trend is in the expected direction (P= 0.031, t = -0.1.86, df=2982; slope subjective = -0.0353 +/- 0.0081; slope for objective = -0.0096 +/- 0.0112). [Note to reader, in prior analysis this slope effect was not significant, with the modified data it now is]

Table 5 Correlation and partial correlation analysis of age, religiosity, educational attainment, and GM score. The values above the diagonal are the spearman rho values for each comparison. Those below the diagonal are the

same pairwise partial correlations controlling for all other variables. All values in bold are significant (at under 0.05). N=2051.

	Education	Age	Religiosity	Politics	OSD	GM
**Education**	-	**-0.14**	0.04	**-0.26**	**0.10**	**0.05**
**Age**	**-0.10**	-	**0.20**	**0.24**	**0.09**	**-0.11**
**Religiosity**	**0.11**	**0.18**	-	**0.17**	**-0.07**	**-0.04**
**Politics**	**-0.24**	**0.19**	**0.14**	**-**	**-0.07**	-0.03
**OSD**	**0.10**	**0.14**	**-0.08**	**-0.06**	-	**0.07**
**GM**	0.03	**-0.10**	-0.02	0.02	**0.080**	-

These results as regards OSD/valence thus largely replicate a prior study [4], excepting that the more extreme rejectors of GM technology are not the least knowledgeable, they are simply less knowledgeable than the extreme acceptors. We return to this issue in Part III.

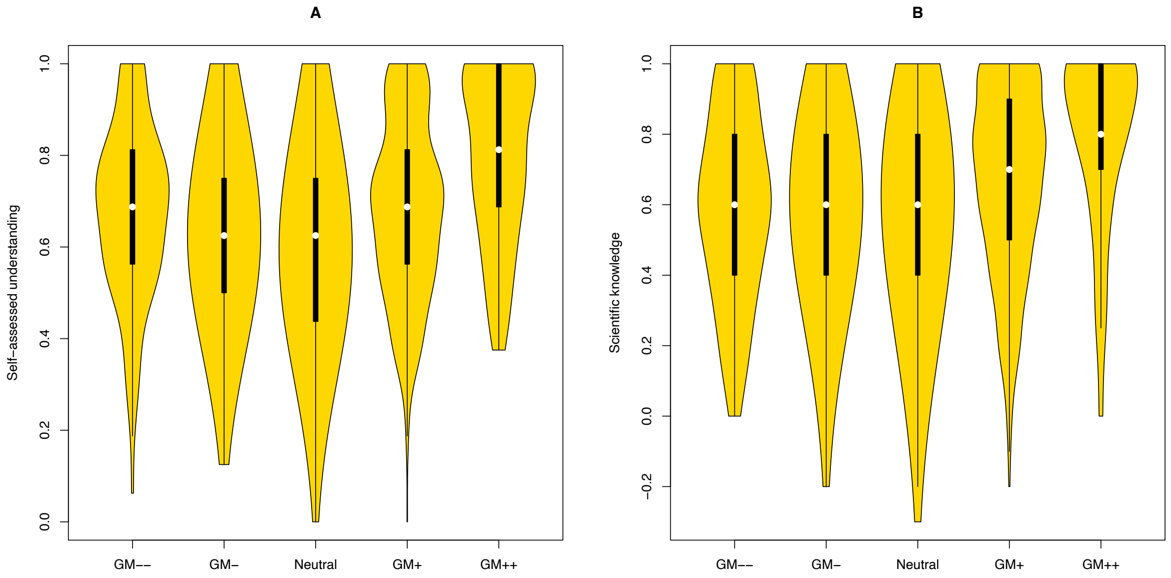

Figure 4. The relationship between position on GM and A) subjective understanding and B) objective knowledge

Replication 2, vaccine attitudes: Given that this survey was performed during the pandemic, with the vaccine roll out having commenced in the UK 5 months prior, we chose to examine behaviour rather than asking about attitudinal position. We asked correspondents whether they had taken the vaccine, intend to but haven't yet, have no intention to, or prefer not to say. The structure of this question is such that we cannot evaluate attitude strength issues.

We observe heterogeneity between the four groups in their subjective understanding (Fig 5A, anova P=0.0159). However post hoc tukey tests reveal differences between those preferring not to answer and those who will be but aren't yet vaccinated exclusively (P=0.036). Those rejecting

the vaccine are no different to those who have taken it (P=0.85) and those who will (P=0.95). By contrast, with respect to knowledge, there is again heterogeneity (Fig 5B, anova, $P=2.4 \times 10^{-5}$) with significant differences between those not getting vaccinated and both groups that will (Tukey post hoc P<0.001 in both cases). Indeed, the only two comparisons that are not significant are between those who will not and those who prefer not to disclose (P=0.98) and the two groups that will or have had it (P=0.85). As then expected, given these results, those rejecting the vaccine have a larger OSD than either group of vaccine acceptors (Fig 5C. anova: $P=6.7 \times 10^{-5}$: Tukey post hoc P<0.003 for both rejector v acceptor comparisons – note no other comparisons are significant). For full Tukey statistics see S Table 2 [given below]. We conclude that the OSD predicts vaccine acceptance, this replicating prior analysis [5, 6].

COVID vaccine hesitancy is centred on the younger age groups (mean age of those who have or will take the vaccine if offered = 49, of those declining = 38, Mann Whitney U test, $P=6 \times 10^{-9}$). There is no difference between those who will or have taken the vaccine and those who say they will not in either educational attainment (Mann Whitney U test, P=0.18) or in religiosity (Mann Whitney U test, P=0.34) although those refusing the vaccine have a relatively high proportion without educational qualifications. In political identity there is heterogeneity between classes (ANOVA, $P=2.5 \times 10^{-8}$) but only two pairwise comparisons are significant: those who have had the vaccine are more conservative than those who will have (Tukey test, P<0.00001) and those who have yet to be vaccinated but will be are more left wing than those who will not have the vaccine. For frequencies see S2A Fig, for sex ratios see S2B Fig (there is no heterogeneity chi sq=5.4, P=0.14, df=3), for educational attainment see S2C Fig for political identify see S2D Fig.

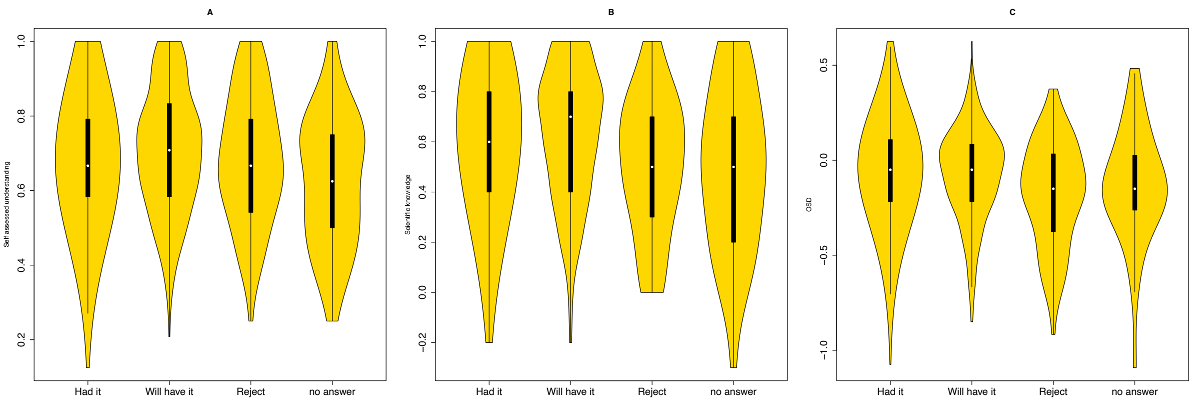

Figure 5. Vaccine stance and A. Subjective understanding, B. Scientific knowledge and C. OSD. For statistics see S2 Table.

Part III Heterogeneity of demographic predictors of OSD

For all four questions, we report that stronger negativity of attitude is associated with a larger OSD. Were there a simple underlying cause, one might expect that all four might show similar underlying demographic predictors. This appears not to be the case. As regards the COVID vaccine the strong predictor was age with younger subjects being more negative/hesitant. As the risk-benefit calculation in this incidence covaries with the age profile, this we suggest is an understandable outlier (no other question revealed a significant age effect in this direction).

Perhaps then, the more intriguing question is why the more abstract Hype and Trust questions – which have the same demographic predictors – seem to diverge from the GM question. That the GM questions might be exceptional is suggested by the fact the Hype and Trust responses correlate with each other much more robustly than either does to the GM question (spearman rank correlation: Hype v Trust, rho=0.44, GM v Trust 0.19, GM v Hype 0.18: for all, $P<10^{-15}$). This suggests that asking specifically about GM is associated with a somewhat different response.

Several further differences between the GM attitude questions and the non-specific Trust and Hype questions are notable. First, there are many more (~5%) in the strong rejecting class than in the prior two strong rejector classes, these being 1-2% (S1A Fig). Second, this class has a ~50:50 sex ratio, different to that for the other two (Fig 6). Third, for the GM question, education attainment is a much weaker predictor of attitudinal position and is indeed not significant on partial correlation analysis (Table 5, see also S1B Fig) nor on multitest correction. Further, unlike the prior two questions, age is a strong predictor (older people are more opposed to GM), before and after partial analysis (Table 5). Fourth, GM attitude is not correlated with political identity (as previously found [1]) while for both Trust and Hype, there is a tendency for those more oppositional to science to be more right wing (Table 2). Fourth, when we restrict analysis to cases where the attitudinal score is less than or equal to zero, we see no significant difference in the slopes relating knowledge score and subjective understanding to attitude for the GM issue [note this result no longer holds]. Fifth, many fewer of the 10 science knowledge questions are predictive of GM position than seen for the two more abstract questions and correlations are generally much

weaker (mean rho for Trust = 0.09, Hype =0.12, GM=0.059: S Table 2). [note that S Table 2 is unaltered in the values for the 10 questions]

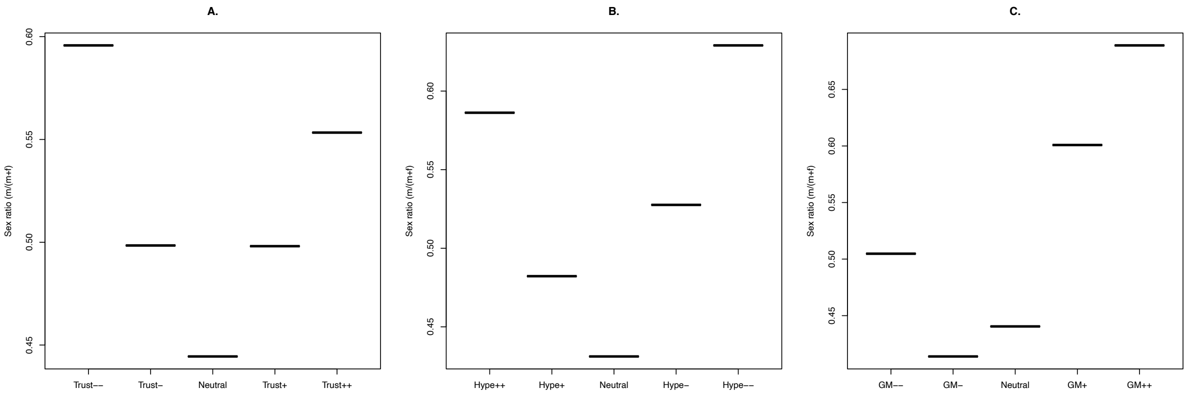

Fig 6 Sex ratio as a functional of attitudinal position for a) Trust, B) Hype and C). GM benefits. All are plotted with more negative views to the left. In both instances there is significant heterogeneity in the sex ratio between the attitudinal groups (Trust: chi squared = 10.9, df=4, P=0.028; Hype: chi squared = 27.6, df=4, P=1.5 x 10^{-5}; GM chi squared = 58.4 df=4, P= 6 x 10^{-12}). Men are more likely to adopt more extreme attitudinal positions for Trust and Hype but only extreme positive attitudes for the GM issue.

A possible explanation for some of this difference is that amongst those most negative to GM is, in addition to those being negative as regards Trust and Hype, a subgroup of older well-educated individuals taken from all political persuasions. A coherent model then, is that there exists a group of sceptics whose responses are not technology specific (revealed by the Hype and Trust questions) and a further demographically different group that are concerned by specific technologies. Consistent with this, employing the dip test [24, 25], there is a significant tendency towards non-unimodality of knowledge scores in the GM strong rejectors (P= 0.00109), but not otherwise (S3 Table). [note this result no longer holds: all are significant except Hype]. This could in turn explain the educational profile (S1B Fig) and weak correlation with educational attainment that isn't robust to covariate control as this larger pool is well educated. In addition, when we analyse which individuals report strong negativity in any of the three parameters (Trust, Hype and GM), we find considerable overlap between the Trust and Hype groups and a large population (92 of 105) of the GM rejectors that do not report strong negativity as regards the Hype and Trust issues (Figure 7).

The intersection between Hype and Trust is highly deviated from null (Z>20), while that between GM attitude and the other two is less deviant from null (Z in range ~4-6) (Table 6). While the size of the three-way intersection (N=3) is modest in absolute terms it is highly significant (Z>16, Table 6). The data thus support a model that the Hype and Trust questions are capturing a generalised antipathy towards science that is independent of specific technology, while negativity as regards the GM question recruits from both this pool of those generally anti-science but mostly those specifically anti-GM. For those showing both negativity as regards GM and the Hype/Trust questions, objections to GM based on technology alone may be considered a smokescreen concealing an underlying antipathy. Given these differences, we caution that the trends that we have observed, especially as regards covariate predictors, are not robustly generalizable. It also implies no universality to predictors of OSD as assayed via similar, but different, questions. It also suggests that there is a subgroup that object to GM but also object when no technology is referenced for which any technological defence may well be a smokescreen. However, for most GM objectors the smokescreen hypothesis doesn't hold weight.

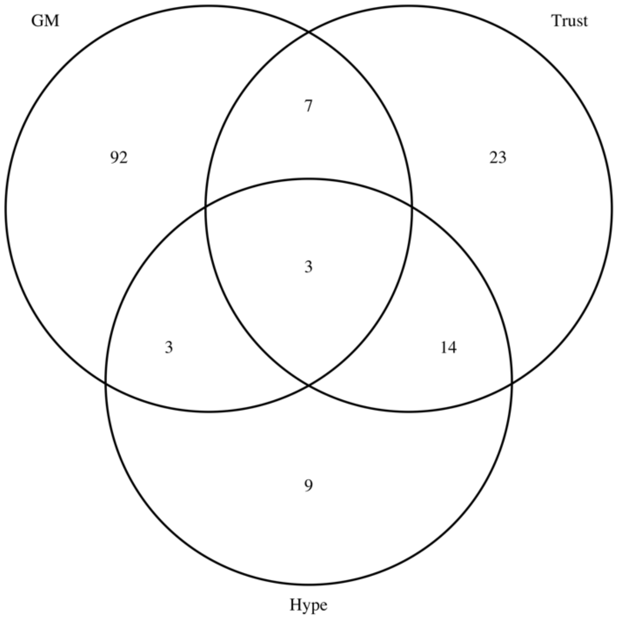

Figure 7 Venn diagram of numbers in each group expressing strongly negative attitudes to three questions.

Table 6 Statistics for the two and three-way intersections as regards numbers expressing extreme negative views towards GM and as regards Hype and Trust issues (i.e. scoring -2 in any of these questions). N1, N2 and N3 are the total numbers in each class. In the two-way interactions, the first named in the comparison column is N1. The intersection is the number of individuals in both groups. P was determined by randomization. Here from the source data three random sets were generated of sizes 29, 47 and 105, to match observed numbers in Hype, Trust and GM respectively. The size of relevant intersections (individuals shared/intersecting in the groups) was determined for each randomization. This was repeated 100,000 times to generate, for each comparison, a distribution of the

number of expected intersecting under a null model of no correspondence. From this vector we determined how many had as great or greater than the observed intersection. P was then given as this number / 100,000. To further assess magnitude of deviation from null we determined the mean of the vectors of numbers intersecting under null and the SD of the same vector. From this we could derive Z = (observed intersection – mean of randoms)/SD of randoms.

Comparison	N1	N2	N3	Intersect	Mean_intersection_rand	SD_rand	Z	P
**Hype v GM**	29	105	NA	6	1.4279	1.1564	3.9538	0.00225
**Hype v Trust**	29	47	NA	17	0.6501	0.7933	20.6112	0.0000
**Trust v GM**	47	105	NA	10	2.3001	1.4678	5.2461	1E-04
**Three way**	105	47	29	3	0.0316	0.1779	16.6843	0.0000
