# Supplementary material for: People with more extreme attitudes towards science have self-confidence in their understanding of science, even if this is not justified
Source: PLoS Biol. 2023 Jan 24;21(1):e3001915. doi: 10.1371/journal.pbio.3001915 (PMC10045565; doi:10.1371/journal.pbio.3001915)
Supplement: Attachment — Submitted filename: Response to reviewers_JP.docx [file pbio.3001915.s012.docx]

Reviewer #1:

In the current manuscript, the authors ask if UK individuals' subjective understanding of science and related topics predicts their attitudes on those topics and technologies. They present data supporting this idea, finding that people have higher levels of subjective knowledge when they have more extreme attitudes, be they supportive or oppositional. They aim to make a contribution by pointing out the positive correlation between subjective knowledge and attitudes at both ends of the attitudinal distribution, and by replicating these relationships when the attitude object is genetic science and scientists more generally, instead of specific genetic technologies.

First, I would like to commend the authors on a clear, well-written, well-evidenced paper. The topic is timely and the empirical findings are of interest to both scientists and laypeople, both inside and outside of the biological sciences.

I have one main critique, which I discuss first. Then I will make several comments to elaborate on the main critique, all of which are numbered point 1, point 2, etc. for easy tracking. Beyond the main critique, the order of comments should not be taken to indicate degree of importance. I conclude the review with how I believe the manuscript could be improved.

The main critique is that the magnitude of the contribution is a bit stretched. The idea of testing whether "extremists" on both the positive and negative sides of the attitude spectrum are more confident in their understanding is important, since much of the prior research on the topic has focused on people whose attitudes oppose those supported by many scientists. This is the biggest contribution of the paper, in my view.

Response: we too agree that this is the most important/novel contribution. We hope the referee agrees that this is well captured in the abstract, our main conclusion being:

*“These results suggest a potentially general model to explain why people differ in their degree of acceptance or rejection of science, this being that the more someone believes they understand the science, the more confident they will be in their acceptance or rejection of it.”*

We have reworked the abstract to ensure that this result is seen as the focal result. In addition, we have altered the title:

*“Why people have different attitudes to science: more extreme attitudes are associated with high self-confidence, even if this isn’t justified”*

However, this contribution is weakened by a few things. The first (point 1) is that although this paper aims to extend the work of Fernbach et al. (2019) and others by examining the subjective understanding-attitude relationship on a bipolar subjective knowledge scale, the scale itself has only five points (fewer than Fernbach et al.'s seven).

Response:
It is indeed the case that for subjective understanding we use 5 points while Fernbach et al use 7. The more important difference between the analyses, however, is that our 5 points are symmetrical around a neutral position while those of Fernbach et al are not. Indeed, as we noted, the Fernbach study, impressive as it is, is focused on understanding those most negative (as is Light et al). We think our important contribution, in considering the matter symmetrically, is that we can determine whether attitude strength predicts both extreme antipathy and extreme acceptance while inviting potentially symmetrical answers.

There are also technical questions as to whether, if one permits a neutral stance, having three divisions on either side would have permitted robust analysis – we think people can know if the oppose or strongly oppose, but can they robustly report whether they weakly, moderately or strongly oppose? We aren’t sure. Either way, the results we find are very robust to analysis changes (and note how we supported our core results with multiple lines of analysis). We now go further and alter the input data to reflect exclusively biology/genetics specific questions (see comment from ref 2) and find all key results to be upheld.

It is true that two of these scale points go beyond the most extreme level of agreement in the Fernbach et al. paper, but the J shape of the relationship is nearly identical to those reported in the Fernbach paper,
as well as in a recent paper in Science Advances (Light et a. 2022) (point 2), which suggests that participants may be distributing similarly across the two scales in the three papers. The fact that the trend is more of a J shape than a true U shape (which is rightly pointed out by the authors in their individual tests of the slopes on both sides of neutral) also slightly weakens the overall claim relying on subjective knowledge to predict attitude extremity, since the relationship is stronger for those who hold opposition attitudes, and opponents are more problematic (societally, scientifically, etc.) than those with attitudes more in line with science (point 3). Here is what is written in Light et al. 2022:

"The subjective knowledge distribution is j-shaped (see the Supplementary Materials for visualizations). Similar to more extreme opponents, those fully in line with the scientific consensus rated subjective knowledge higher than moderate opposers (but lower than extreme opponents). However, whereas the confidence of those in agreement with the established science is substantiated by their actual knowledge, the confidence of extremists appears to be misplaced."

Response: we don’t think that the referee’s reference to a J shaped function in Light et al is especially fair. The parallel analysis to our key result in Light et al is their figure 1 panel 2:

And the lower panel of their figure 2:

For a reminder here is our Figure 1:

Like us, the plots of Light et al consider the relationship between subjective knowledge (Y axis) and attitudinal position (x axis). Clearly those in the main text of Light et al are not U or J shaped functions. Indeed, the figure legend states that opposition is positively associated with objective knowledge. In the main text they state: “*The relation between opposition and subjective knowledge is positive for all issues but is not statistically significant for climate change, Big Bang, or evolution (P = 0.13, 0.94, and 0.55, respectively).*”

What the referee appears to be referring to are some figures in the supplement. Figure S5, related to a manipulation experiment and so not comparable to our study, appears to be J shaped. However, their figure S8 (for study 5 concerning covid mitigations) is not obviously J shaped. Their figure S2 (related to their studies 1 and 2) appears to be J shaped. We don’t however see the relationship between their main figure 1 central panel and fig S2 – if we understand correctly, they are the same data but one plot is clearly implying a linear effect – the one in the main text. In fig 1 of Fernbach et al 2019 there may also be a J shaped distribution, but this includes a class (class 1) with no opposition that has a lower subjective score than class 2.

Additionally, we think here the referee may have misunderstood our results to think that “*the relationship is stronger for those who hold opposition attitudes*”. The referee thinks the relationship between attitude strength and subjective understanding is stronger for those with a negative disposition (Trust <=0, Hype <=0) than those with a positive attitude (Trust >=0, Hype >=0). Our data importantly report the opposite. We wrote:

“*Given this improvement of the quadratic fit, we additionally consider the correlation between subjective understanding and attitude score for both halves of the distribution separately (Prediction 1c). This confirms the trends (Trust >= 0, rho = 0.27, P=2 x 10-16; Trust <= 0, rho = -0.13, P=8.7 x 10-6; Hype >=0, rho = 0.33, P = 2 x 10-16; Hype <=0, rho =-0.17, P=9 x 10-10).* *We conclude that as subjective understanding increases so too does attitudinal extremity, in both positive and negative directions*.”

Notice that the rho values for those with positive attitudes (Trust >=0, Hype >=0) are approximately double those for the sceptics. In our mode of analysis, the self-confidence much better predicts the strong acceptors.

Taken together, in short, we don’t think a fair reading of Light et al (or prior data) would consider that their main claims and main figures (above) pre-empt our report of a U shaped function. We would like to note that the journal has permitted our discussion of the paper of Light et al to be covered under their 6 month anti-scooping policy. However, we are not sure that this is entirely necessary as, given the plots above, we feel that we have established a novel result, even if one might suggest that it is related to one hidden away in the supplement of this prior paper.

To address these issues we now add:

*One might question, however, why the trend that we see has not been reported previously. Recent comparable analyses, notably that of Light et al. [6], for example, report a monotonic relationship between degree of opposition to science and subjective knowledge (see their figures 1 and 2), with those most opposed with the highest subjective knowledge. One likely reason that they do not detect the U-shaped function that we observe is that they assay degree of opposition, while we present a symmetrical set of alternatives thus assaying degrees of support and opposition. However, while their main figures and analysis are not supportive of the trend that we observe, it may be the case that some of Light et al’s data is supportive of higher subjective knowledge of those less opposed. Closer scrutiny of the supplementary figures of Light et al [6] suggests that at lower degrees of opposition, at least for some issues, there may be raised subjective understanding, what they refer to as a J shaped curve: this is seen in their supplementary Figures 2 and 5, but not in their Supplementary Figure 8, although no statistical analysis was performed. Even if they do detect the same effect, in our data the strength of association between subjective understanding and attitude is stronger for those with a positive attitude than those with a negative attitude. This is not supported by their data, possibly again because of their scoring system being focused on degrees of opposition.*

We hope that this provides fair clarification of the position of our data with respect to the claims of Light et al.

A small additional point here is that there appear to be very few respondents at the extreme agreement end of the attitude distributions (point 4). In other words, while useful and interesting, the absolute value approach may obscure the result that most of the action appears to be happening on the opposing side of the scale.

Response: we were aware of the rarity of the extreme opponents early in our analysis and this indeed informed the statistics used. A methodology that compares means, for example, with no account of sample sizes has a strong potential to mislead. For this reason, all analyses feature all data rather parametric captures.

Another reason that I believe the magnitude of the contribution to be a bit stretched is that the authors do not discuss the scientific topics both in Fernbach et al. 2019 and Light et al. 2022 where the relationship between subjective understanding and attitudes was near-zero or not statistically significant. They were climate change, the Big Bang, and Evolution (point 5). It is possible that the authors of the current manuscript intended the paper's generalizations regarding subjective understanding to apply only to a specific subset of scientific issues relating to genetics, in which case this critique may be misplaced and the authors are justified in not discussing those findings, but this could be helped by adding some clarifying writing on the point (and perhaps editing the manuscript title as well).

Response: this is an excellent point. We thought we had addressed it to some degree. We started by showing that our data does give a positive knowledge attitude relationship (indicative of non-polarised science) and in the caveats section we wrote:

“*For our survey data we recover the classical positive correlation between knowledge and attitude [3]. Whether results extend to cases where high educational attainment is associated with both strongly positive and strongly negative attitudes [1, 2] is unknown.”*

However, we can see that this is a key caveat and deserves more attention. To this end we now lead the caveats section with this:

*These results come with numerous caveats the most important of which question the generality of our results. For our survey data we recover the classical positive correlation between knowledge and attitude (Supplementary results 1) seen in cases where attitude to science is not affected by religio/political factors [3]. Whether our results extend to cases where high educational attainment is associated with both strongly positive and strongly negative attitudes [1, 2] is unknown. Indeed, these instances tend to be those where religio/political positions appear to affect attitude to the aligned science, for example climate change in the case of political polarization, evolution and big bang in the case of religion [1]. Whether attitude strength in our measure is predicted by strength of subjective understanding in such contexts remains to be seen, although Light et al. [6] report no such effect when considering evolution, big bang and climate change using their scoring system. More generally, while we could allow for religiosity in our analysis, we have no measure of political polarization. While attitudes to GM tend not to align with religio/political inclinations [1], it remains to be seen whether our non-technology focused questions are also unaligned.*

We also tone down our claims – we talk about a *potentially* general model not a general model.

I was also a little confused when the authors discuss the implications of the Hype and Trust analyses. Because these items focused on genetic science and scientists more broadly, the authors interpret finding the same pattern of results as the more specific measures as evidence that objections based on specific technologies may be "a smokescreen hiding a more general antipathy." This may or may not be true, depending on what the authors meant. It is my hunch that the Hype and Trust measures are actually capturing more extreme participants—being broadly anti-science is more extreme than being anti-GM (point 6).

Response: this is our inclination too -see below.

So, someone who disagrees with those may not act the same when asked about a specific technology, which they may not evaluate in the context of where the technology sits among their larger anti-scientific attitudes or identity. This is speculation on my part, but it's also an empirical question that the authors could test. So, while it is true that people who are
broadly anti-science are likely to also be against specific technologies they view as "scientific," this does not mean that all those against a specific scientific technology are broadly anti-scientific, which is how the current Hype and Trust implications read to me. It's an important distinction—have you uncovered a broad psychological phenomenon or another specific instantiation of the phenomenon among a more extreme population?

Response: this is a helpful point. As we noted, the anti-GM constituents include the extreme general opponents and a separate group of well-educated objectors. Thus, there seems to be a constituency of well-educated anti GM folks who are not broadly anti-science, but also a subgroup who are broadly anti-science as revealed by the technology independent questions. To further examine this issue, we add a new section considering the degree of overlap between those most negative in the three attitudinal questions (GM, Hype and Trust). We show that the degree of overlap is highly significant on Monte Carlo simulation but that most GM objectors are not the strong Hype Trust rejectors. The Hype -Trust overlap is extremely large (Z>20). The new analysis is in Part III with a new Figure 7 and Table 6. We hope this answers the questions.

I do not believe it is the role of reviewers to decide whether a manuscript is suitable for publication in a specific journal (that is the job of the editors), so I will instead provide some thoughts on how I believe the paper could be improved.

a)      Focusing on the bipolar nature of the subjective understanding - attitude relationship.

Response: we agree – see new abstract and title. We also finish the introduction with a broad statement of novelty:

*We report two novel results. First, we observe that individuals with more extreme attitudes, in either positive or negative directions, tend to be more confident that they understand genetic science. This provides a potentially general underpinning model for attitudinal strength, the generality of which is yet to be determined. Second, while replicating the finding that those reporting negative attitudes are mistaken in their belief that they understand the science, we find this to be observed both with and without reference to specific genetic technologies. Objection to specific technologies may thus for some be a smokescreen concealing an underlying negativity underpinned by unjustified self-confidence. We discuss implications of these results for effective science communication and more broadly.*

b)      Focusing less on the manuscript's current replications of prior work, and being clear about what is new.

Response: While we agree with the need to focus on the novel core results, we slightly disagree about the importance of the replication studies. We were remiss not to explain the importance of the GM and vaccine tests. These are more than replications. If these trends were not seen in our current data then that would influence the interpretation of our core results. We now differently motivate the inclusion of this material to go beyond mere replication. We write:

*In part IIb, we seek to broadly repeat the result that OSD predicts attitudinal position as regards GM (replication test 1) and vaccines (replication test 2) [4-6]. The structure of the GM question also enables us to address the attitude strength issue. These tests are important in two regards. First, to see whether the over-confidence effect [4-6] generalises to technology non-specific questions, it is also important to see that within our data we see the technology-specific effect. Were this not the case then the relationship with prior claims would be uncertain. Second, the prior claims are relatively new and their resilience to replicational studies uncertain although recent work supports strong generality across multiple scientific issues, but not all [6].*

c)      Doing so in new studies with larger, nationally-representative samples from the UK or elsewhere in order to make sure there are enough participants at the extremes to have confidence in the bipolar findings. Also including additional scientific topics if the authors intended for their findings to be more broadly generalizable.

Response: we did indeed look to see if any other survey might work with our questions but couldn’t find one. This is not surprising as subjective understanding is a very recently introduced variable. We hope our contribution is strong enough as is, caveats permitting, not least because we find the same trend for three distinct questions. We profoundly hope that our work will stimulate others to provide related downstream analyses to address the nature of extremity both positive and negative.

d)      Editing the writing to be more specific so as to ensure that the magnitude of the contribution and intended generalizability of the findings does not come off as overblown.

Response: we agree – we hope the expansion of the important caveats addresses this issue.

e)      Providing histograms of the distributions of participants over attitude levels for all studies. This is a smaller ask, of course, but I could only find them for the Trust and Hype measures.

Response: They were there (and still are): Fig S1A and Fig S2A.

Best of luck to the authors, and thank you for working on this important topic.

Response: we thank the reviewer for their thoughtful and constructive analysis of our manuscript


Reviewer #2:

This is an interesting paper on an important issue, one I have struggled with in more than one study. The key question of the paper is whether attitudinal strength is predicted by subjective understanding (p. 5 of the manuscript); the fact that it is in the domain of genetics is an important result.

Response: we are pleased that the referee sees the importance of this novel result

To my mind, the many subquestions the authors pose are informative, and the statistical tests used to examine them seem valid. My primary complaint is that the methods are opaque enough that assessment of the work is difficult and competing explanations cannot be ruled out. I also urge caution in the concluding recommendations.

(1) The dependent variable is described as the "modulus" of the Hype and Trust scales (p. 5). I was not sure what this means and asked a mathematician, who agreed that the authors probably meant "absolute value." The R code confirms this, but this critical detail should be stated outright in the manuscript.

Response: modulus and absolute are interchangeable terms. On first use we now introduce both. We are grateful that the referee consulted the R code to check.

(2) One independent variable is described as the mean of six normalized (z-scored?) scales meant to capture subjective understanding. For two of the six questions, I couldn't even hazard a guess as to the response scales—the manuscript only says "Questions 1 and 2 invited a response in one of four ways and were graded 1-4 (4 maximum)." What are these ways?

Response: this was an error – they are now graded 0-3. After we normalise by dividing by three the range is 0-1. In the original version it was 1-4 normalised by 4. As this doesn’t give a minimum of zero, we have reworked all the stats in the main text to reflect the 0-3 change. As you might expect there is no material change (see pdf of manuscript with changes marked). We also note that, while one might also question this scoring system, results are robust to removal of these two questions – see point below.

The response options are in the supplemental materials, but for a key variable, this information should not be buried so deeply. Moreover, even once I dug them up, it was not clear how the questions were in fact coded. For the "well-informed" question (Q1112), I assume responses were reverse coded. If that's right, the manuscript should say so. For the "understanding-science-in-the-news question" (Q1111), I really can't say—were they reverse coded, or reverse-coded omitting option 4 "I don't see or hear science news stories," or something else? I could not find this information in the R code; either I missed it, or this step occurred prior to the analysis code.

Of course, these details probably do not affect the big picture, since the measure used in the analyses is a mean over all scales, and these scales showed good internal consistency. But the lack of clarity on these details will confuse the reader trying to follow the logic of the study.

Response: we can only apologise for our lack of transparency here. The data was pre-processed and we should have included the pre-processing scripts too. We now provide these so anyone can run from the original questionnaire to the input data files to the analysis. We also now specify more in the manuscript as this is an important variable. For all questions after coding (0-4 or 0-3), the data was normalised to a maximum score of that question (i.e. 3 or 4). The score thus has a scale from 0 – no self-confidence to 1, maximum self-confidence.

(3) It is never explicitly stated why the authors chose the covariates that they did. It wasn't clear to me from the supplementary materials whether the Public Voice survey measured other demographic characteristics, but political ideology is a conspicuous possible confound. If age, religiosity and education were the only available covariates, the manuscript should say so; if they were chosen for particular reasons, the manuscript should state them.

Response: We now provide a fuller defence for the covariates. Unfortunately, political attitude is not a covariate available to us. We write:

*Both educational attainment [1] and religiosity [18] are known to relate to science attitude. As age may well covary with both, and is likely to be relevant in the context of COVID, where the old were especially affected, we also include it as a covariate. We have no score of political extremism.*

(4) The other independent variable is clear enough in how objective knowledge is operationalized. But it raises another quite general, conceptual problem with the measures used, one that some of the papers the authors cite also struggle with. Call it the domain congruence problem.

In Replication 1 (p. 13), the authors report the same relationship between the objective/subjective deficit and attitudes towards genetically modified foods that were reported in Fernbach, Motta, etc. The trouble is that the OSD is the difference between an objective knowledge score and a subjective knowledge score, neither of which exclusively concerns genetics. It is true that the objective knowledge measure contained a number of basic science facts that sit squarely in the domain of genetics, but it contained plenty that arguably (viruses, dinosaurs) or inarguably (electrons, radioactivity) do not. The low internal consistency of these scales, while unsurprising for an objective knowledge measure, is surely an opening for the critic of the authors' approach. How is the reader to know that the main results are not an artifact of averaging over all of these measures, only some of which have to do with genetics? In other words, rather than people who most oppose genetics
knowing relatively little about genetics, it could be that people who most oppose genetics know relatively little about science-y stuff.

Response: this is a good point. There are certainly two knowledge questions that are not even in a broad genetics remit. Likewise, two of the subjective understanding questions (1 and 2) were more generic. We have repeated all of the analyses using only those questions that may be considered biological/genetical and present these results as a supplement results 2 – this is a mirror of the original paper but with all tables, statistics and figures updated. As can be seen the core results are unaffected. We also now comment:

*“In addition, while we have focused on public attitudes to genetics, not all of our science knowledge questions were biological. Likewise, two of our subjective understanding questions concerned science more generally. Might our results be misleading for not focusing more on biological/genetical questions? To address this concern, we have reanalysed the data this time defining “scientific knowledge” employing exclusively the questions that were biological (ie. omitted those about radioactivity and atoms) and defining subjective understanding by the 4 questions that were explicitly genetical (i.e. questions 3-6, see Methods). The main conclusions are unaffected (Supplementary materials 2) but we observe three qualitative changes to the results (meaning a switch from significance to non-significance or vice versa). These are 1: the partial correlation between modulus of trust score and educational attainment is now marginally significant – but not after multiple test correction; 2. In the context of GM attitudes when we restrict analysis to only instances where the attitude score is less than or equal to zero, the slopes of the two lines (subjective or objective versus attitude) are now significantly different, whereas before they were not, although the trend was in the expected direction (new results: P= 0.031, t = -0.1.86, df=2982; slope subjective = -0.0353 +/- 0.0081; slope for objective = -0.0096 +/- 0.0112). This strengthens the evidence that those rejecting GM technology have higher subjective understanding than objective knowledge. 3. For the Hartigan and Hartigan dip scores the distribution of knowledge scores for those most negative in attitude is significantly non-unimodal in all cases except Hype. Previously, GM was unique in this regard. We conclude that our core findings are resilient to, and to some degree strengthened by, restriction of questions that are exclusively biological/genetical.* “

The subjective knowledge measure has the same problem—some questions are straightforwardly about genetics, others are about how well people feel they understand science news—although in that case, internal consistency was high.

Response: as noted above we have redone all analyses removing the non-genetic questions.

If, for example, it turned out that (a) the effects the authors report are being driven entirely by the subjective knowledge questions about science news and (b) the response option "I don't see or hear science news stories" was included as the endpoint of the "understanding-science-in-the-news" question, meaning the lowest degree of subjective understanding is assigned to people who don't pay attention to science news at all, then the main analyses do not reflect a relationship between subjective understanding and attitudes, but rather between consumption of mainstream science news and attitudes. This is a quite different phenomenon than the one the paper is meant to address, and the brief note in the Discussion about trust in media sources further supports this alternative explanation. The same problem applies to COVID subjective and objective knowledge, of course.

One way to address this problem is to test for the same patterns in the data using only the subscales that are unequivocally about genetics (or COVID). This may seem like a chore, but I raise the issue because Light and colleagues have been understood as claiming that dumb people oppose science and smart people support it. I believe the picture is more complicated than this, and the current paper deepens our understanding of the complex interplay between attitudes, subjective knowledge, and objective knowledge. But if the authors wish to avoid also being understood as saying that dumb people oppose science, they should address the domain congruence issue with further analysis or additional discussion.

Response: we hope that our re-analysis addresses this. We also now discuss the issue in the caveats sections. We would also like to point out that at no point do we endorse the “dumb” folks don’t get science view. We indeed argue against the Dunning Kruger interpretation and note that it is possible to be logically smart but if handling the wrong evidence, come to the wrong conclusion. We also now present the correlations between all science knowledge questions as this we think reveals an interesting truth. We have expanded this to clarify:

*In previous studies, authors speculate [4, 5] about Dunning-Kruger type effects [10], whereby low knowledge may be associated with a low analytical abilities that prevent understanding that one’s knowledge is limited. We didn’t measure variables relevant to ascertainment of this specific hypothesis. Nonetheless, further dissection of our 12 factual questions suggests that such a model is insufficient. For both Hype and Trust the three questions of the 12 true/false “textbook” questions most strongly correlating with these two attitudinal positions (S Table 1) are questions 4 (‘By eating a genetically modified fruit, a person's genes could also become modified’), 5 (‘All radioactivity is human-made’) and 8 (‘Tomatoes do not naturally contain genes - genes are only found in genetically modified tomatoes’). In addition, answers to these three questions were strongly correlated (Table S4). These are the only three questions that unambiguously address human interventions (or lack thereof). This suggests that the effect might be construed as more conspiratorial in nature. A conspiratorial aspect is also attractive in so much as it goes some way to explain the negativity [5]. This need not imply a lack of logical inference skills. Indeed, that those who consider that only GM tomatoes have genes also consider that it possible to take up genes from GM crops is consistent with a model of logical inference from incorrect understandings.*

(5) With full recognition that specifying the practical implications of these findings is extremely difficult, I submit that "asking people to explain their understanding of the science" (p. 20) will almost certainly not help. I work with a number of practitioners in public health and health communication, and I have yet to find one that believes making others feel stupid is a good approach to softening attitudes toward policies or interventions that the best science suggests are safe and effective.

Response: the referee’s experience here is extremely valuable. We somewhat miswrote this section leading to possible misinterpretation. We agree that making individuals feel stupid is not the way to go as regards that individual. We indeed noted that the prior claims were not replicated. We now add that this may well induce a backfire effect. Our speculation was different – that when others are listening to arguments getting someone to explain the science might be helpful as this could reduce their currency in the eyes of others. This is what we now write:

*Second, these results suggest an alternative approach to tackling science denial/rejection, one that focuses on OSD [4] rather than, or alongside, gain in knowledge/understanding. Training to improve logical reasoning skills helps correct similar objective subjective deficits [10]. It has been proposed that asking people to explain their understanding of the science may also help, as failure to explain the underlying mechanisms disturbs the sense of understanding [30, 31]. The claim [31] that this can lead to moderation of position could not, however, be replicated [32], although other evidence suggests that people are more likely to change their attitudes when their confidence in their knowledge underpinning the attitude is reduced [12]. This strategy also runs the risk of inducing backfire effects [33], whereby individuals become more entrenched when challenged. Whether the focal sceptic changes his or her mind, however, need not be the issue. Instead, prior results [31, 32] suggests that explication of understanding, when understanding is weak, leads to reduced confidence in that understanding. Thus, a debating strategy would be to ask those most sceptical of the science to explain their understanding of the science. They commonly will be unable to do so [31, 32], which may make their case less persuasive to others. This effect on others is worth experimental investigation.*

Moreover, "friends and family" and "government's scientific advisors" are anything but non-partisan sources, albeit for different reasons. For friends and family, there is damning evidence of homophily (Mosleh et al., 2021, "Shared partisanship dramatically increases social tie formation in a Twitter field experiment"), meaning trusting one's friends and family hardly precludes ideological sorting. Perhaps scientific advisors to the government are still considered neutral in the UK, but they are not in the US; in a recent, qualitative measure probing people's reasons for trusting or not trusting information from public health scientists, those who do not trust it invoked our top COVID advisor Anthony Fauci almost constantly despite the fact that he has not appeared on TV in roughly a year.

What should the authors recommend based on their findings, in that case? I wish I knew, but at the very least I would urge further reflection on this important question.

Response: the referee’s points are very well taken. However, we don’t wish to go down this road not least because we think the referee is right – in the UK there is good trust in science advisors who are mostly seen as neutral. We understand the same is not seen in the US. This makes broad brush recommendations along such lines inadvisable. We have reworded to be more cautious.


Reviewer #3:

Why people have different attitudes to science: it isn't just about what you know but what you think you know

Cristina Fonseca, Jonathan Pettitt, Alison Woollard, Adam Rutherford, Wendy Bickmore, Anne Ferguson-Smith , Laurence D. Hurst

The abstract is good, and explains why the studies are important and of general interest.

In general, I think this paper makes a definite contribution to the field, in terms of examination of both positive and negative attitudinal extremes, and examination of the generality vs specificity of the question focus.  The analysis seems quite sophisticated.   I found it very dense, with both the analysis and the style often hard to follow.  This may be more a function of my limitations, rather than a problem with the paper.

Response: we thank the reviewer for their positive comments.
