# Supplementary material for: People with more extreme attitudes towards science have self-confidence in their understanding of science, even if this is not justified
Source: PLoS Biol. 2023 Jan 24;21(1):e3001915. doi: 10.1371/journal.pbio.3001915 (PMC10045565; doi:10.1371/journal.pbio.3001915)
Supplement: Attachment — Submitted filename: PLOS Bio Round 2.docx [file pbio.3001915.s013.docx]

I served as Reviewer 1 on the previous round of reviews for the manuscript. In my opinion, the authors have done a good job of addressing reviewers’ comments in good faith, and have made important improvements to the paper. However, I still hold three of my earlier concerns about the paper, which I think are important to address:

1. The novelty of the pattern of results
2. The limited number of observations at the extremes of the attitude distribution
3. Writing regarding the generalizability of the findings

In the previous round of reviews I mentioned that Light et al. (2022) and Fernbach et al. (2019) show a J-shaped pattern in the relationship between subjective knowledge and attitude. Putting aside the Light paper (with the journal’s grace period in mind), I still have this concern. I provide more specifics in my responses to the authors’ writing below. The authors write:

*Like us, the plots of Light et al consider the relationship between subjective knowledge (Y axis) and attitudinal position (x axis). Clearly those in the main text of Light et al are not U or J shaped functions. Indeed, the figure legend states that opposition is positively associated with objective knowledge. In the main text they state: “The relation between opposition and subjective knowledge is positive for all issues but is not statistically significant for climate change, Big Bang, or evolution (P = 0.13, 0.94, and 0.55, respectively).”*

*What the referee appears to be referring to are some figures in the supplement. Figure S5, related to a manipulation experiment and so not comparable to our study, appears to be J shaped. However, their figure S8 (for study 5 concerning covid mitigations) is not obviously J shaped. Their figure S2 (related to their studies 1 and 2) appears to be J shaped. We don’t however see the relationship between their main figure 1 central panel and fig S2 – if we understand correctly, they are the same data but one plot is clearly implying a linear effect – the one in the main text. In fig 1 of Fernbach et al 2019 there may also be a J shaped distribution, but this includes a class (class 1) with no opposition that has a lower subjective score than class 2.*

Ignoring the Light paper, Figure 1 of Fernbach et al. (2019) is the key figure to consider. There, they indeed discuss the results of a linear regression model, but the right panel of Figure 1 (pasted below) is a more specific visualization of the subjective knowledge-attitude relationship. The authors of the current paper under review interpret the figure as non-J-shaped because the “no opposition” group (level 1 on the graph) has a lower mean subjective knowledge score than the level 1.5 group. However, this interpretation ignores the fact that these “extremity of opposition” levels are averages of two variables, and levels 1 and 1.5 have higher extremity means than all levels between 2 and 5, but lower than levels 5.5 to 7. The relationship is very similar to that shown in the current manuscript. The General Discussion in the Fernbach paper says, “All analyses in the main text use linear models, but there is some evidence of nonlinearity (see for example the uptick in self-assessed knowledge for those scoring 1 or 2 on extremity of opposition in Fig. 1). We therefore conducted tests of quadratic effects to provide

a better understanding of any nonlinearities in the data. For self assessed knowledge, in all studies there is a significant quadratic effect of extremity of opposition. For most of the range, the effect of opposition on self-assessed knowledge is positive, but the relationship turns negative at low levels of opposition. Thus, the interpretation based on the linear model—that self-assessed knowledge increases with opposition—should be qualified to acknowledge that this is not true at very low levels of opposition.”

*Additionally, we think here the referee may have misunderstood our results to think that “the relationship is stronger for those who hold opposition attitudes”. The referee thinks the relationship between attitude strength and subjective understanding is stronger for those with a negative disposition (Trust <=0, Hype <=0) than those with a positive attitude (Trust >=0, Hype >=0). Our data importantly report the opposite. We wrote:*

*“Given this improvement of the quadratic fit, we additionally consider the correlation between subjective understanding and attitude score for both halves of the distribution separately (Prediction 1c). This confirms the trends (Trust >= 0, rho = 0.27, P=2 x 10-16; Trust <= 0, rho = -0.13, P=8.7 x 10-6; Hype >=0, rho= 0.33, P = 2 x 10-16; Hype <=0, rho =-0.17, P=9 x 10-10). We conclude that as subjective understanding increases so too does attitudinal extremity, in both positive and negative directions.”*

*Notice that the rho values for those with positive attitudes (Trust >=0, Hype >=0) are approximately double those for the sceptics. In our mode of analysis, the self-confidence much better predicts the strong acceptors.*

The authors are correct that I was misreading the results in this section previously. My apologies.

*Taken together, in short, we don’t think a fair reading of Light et al (or prior data) would consider that their main claims and main figures (above) pre-empt our report of a U shaped function. We would like to note that the journal has permitted our discussion of the paper of Light et al to be covered under their 6 month anti-scooping policy. However, we are not sure that this is entirely necessary as, given the plots above, we feel that we have established a novel result, even if one might suggest that it is related to one hidden away in the supplement of this prior paper.*

I don’t think it’s a fair to say that the J- (or U-)shape pattern/discussion is “hidden away in the supplement.” It’s in Figure 1 and the General Discussion of the Fernbach paper. That paper was also a short report published in a journal with strict length limits, which encourage authors to put a lot of discussion and analyses in the supplemental materials. Again, I believe the findings the authors document are important, but I still believe the degree of novelty is slightly inflated.

I am also still somewhat concerned about the limited number of observations at the extreme agreement end of the attitude distributions. In response to this in the previous round of reviews, the authors wrote:

*[W]e were aware of the rarity of the extreme opponents early in our analysis and this indeed informed the statistics used. A methodology that compares means, for example, with no account of sample sizes has a strong potential to mislead. For this reason, all analyses feature all data rather parametric captures.*

While the authors are correct about statistical tests that ignore sample sizes, both regression or correlational analyses can obscure differences in true relationships for parts of the X or Y variables’ distributions with fewer observations. The authors’ approach (“featur[ing] all data”) is helpful for preserving statistical power, but does not help overcome potential biases due to limited data. As I said in my initial review, I think running a new study that ensures more-or-less equal numbers across the attitude distribution would significantly strengthen the paper’s findings, especially considering that the biggest contribution is testing the relationship using a bi-polar attitude measure (with the novelty on the agreement end). Given the similarity of the shape with Fernbach’s 7-point attitude measure, running a new study like this would also have the added benefit of showing that the data patterns are not the result of some participant scale-use artifact. Another benefit would be to measure participants’ political ideology and control for it, thus addressing reviewer 2’s point on politics.

Finally, in my earlier review I raised a question about how best to interpret participants’ responses on the broader anti-science attitude measures. See my initial writing and the authors’ responses below.

*I was also a little confused when the authors discuss the implications of the Hype and Trust analyses. Because these items focused on genetic science and scientists more broadly, the authors interpret finding the same pattern of results as the more specific measures as evidence that objections based on specific technologies may be "a smokescreen hiding a more general antipathy." This may or may not be true, depending on what the authors meant. It is my hunch that the Hype and Trust measures are actually capturing more extreme participants—being broadly anti-science is more extreme than being anti-GM (point 6).*

*Response: this is our inclination too -see below.*

*So, someone who disagrees with those may not act the same when asked about a specific technology, which they may not evaluate in the context of where the technology sits among their larger anti-scientific attitudes or identity. This is speculation on my part, but it's also an empirical question that the authors could test. So, while it is true that people who are broadly anti-science are likely to also be against specific technologies they view as "scientific," this does not mean that all those against a specific scientific technology are broadly anti-scientific, which is how the current Hype and Trust implications read to me. It's an important distinction—have you uncovered a broad psychological phenomenon or another specific instantiation of the phenomenon among a more extreme population?*

*Response: this is a helpful point. As we noted, the anti-GM constituents include the extreme general opponents and a separate group of well-educated objectors. Thus, there seems to be a constituency of well-educated anti GM folks who are not broadly anti-science, but also a subgroup who are broadly anti-science as revealed by the technology independent questions. To further examine this issue, we add a new section considering the degree of overlap between those most negative in the three attitudinal questions (GM, Hype and Trust). We show that the degree of overlap is highly significant on Monte Carlo simulation but that most GM objectors are not the strong Hype Trust rejectors. The Hype -Trust overlap is extremely large (Z>20). The new analysis is in Part III with a new Figure 7 and Table 6. We hope this answers the questions.*

I appreciate the authors’ additional writing on this point, including clarifications regarding generalizability, and I believe we agree on the interpretation of the data. The Venn diagram (and subsequent statistical tests) demonstrate that the degree of overlap between Hype and Trust and GM extreme opposition is actually quite small (but non-zero and statistically significant). Therefore, I still think that the idea that objection to specific technologies is a smokescreen for more general anti-scientific attitudes is not really supported in the data, especially considering the relatively small sample sizes. I think this could be solved by walking back the claim of a “potentially general model” in the title, abstract, and main text.

One final note: I suspect this is not the authors’ fault, but I was still unable to see the supplementary figures. Is it possible they appeared as links that were broken in my reviewer packet PDF?

Thanks again to the authors for their care and thoughtfulness.
